# Mass spectrometry captures biased signalling and allosteric modulation of a G-protein-coupled receptor

**DOI:** 10.1038/s41557-022-01041-9

**Published:** 2022-11-10

**Authors:** Hsin-Yung Yen, Idlir Liko, Wanling Song, Parth Kapoor, Fernando Almeida, Joanna Toporowska, Karolina Gherbi, Jonathan T. S. Hopper, Steven J. Charlton, Argyris Politis, Mark S. P. Sansom, Ali Jazayeri, Carol V. Robinson

**Affiliations:** 1 Chemical Research Laboratory, University of Oxford, Oxford, UK; 2 OMass Therapeutics, Oxford, UK; 3 Department of Biochemistry, University of Oxford, Oxford, UK; 4 Department of Chemistry, King’s College London, London, UK; 5 School of Life Sciences, Queen’s Medical Centre, University of Nottingham, Nottingham, UK; 6 Kavli Institute for Nanoscience Discovery, Oxford, UK; 7 Present Address: Institute of Biological Chemistry, Academia Sinica, Taipei, Taiwan; 8 Present Address: Rahko, London, UK; 9 Present Address: Faculty of Biology, Medicine and Health, School of Biological Sciences, The University of Manchester, Manchester, UK

**Keywords:** Membrane biophysics, Mass spectrometry, Mechanism of action

## Abstract

G-protein-coupled receptors signal through cognate G proteins. Despite the widespread importance of these receptors, their regulatory mechanisms for G-protein selectivity are not fully understood. Here we present a native mass spectrometry-based approach to interrogate both biased signalling and allosteric modulation of the β_1_-adrenergic receptor in response to various ligands. By simultaneously capturing the effects of ligand binding and receptor coupling to different G proteins, we probed the relative importance of specific interactions with the receptor through systematic changes in 14 ligands, including isoprenaline derivatives, full and partial agonists, and antagonists. We observed enhanced dynamics of the intracellular loop 3 in the presence of isoprenaline, which is capable of acting as a biased agonist. We also show here that endogenous zinc ions augment the binding in receptor–G_s_ complexes and propose a zinc ion-binding hotspot at the TM5/TM6 intracellular interface of the receptor–G_s_ complex. Further interrogation led us to propose a mechanism in which zinc ions facilitate a structural transition of the intermediate complex towards the stable state.

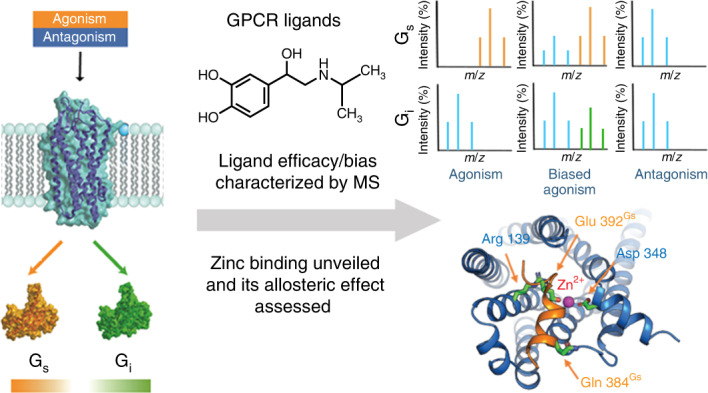

## Abstract (web summary)

Native mass spectrometry has been used to interrogate both biased signalling and allosteric modulation of the β_1_-adrenergic receptor. Simultaneously capturing the effects of ligand binding and receptor coupling to different G proteins has enabled the relative importance of specific interactions to be investigated.

## Main

G-protein-coupled receptors are involved in many physiological processes and as a consequence account for nearly 40% of current drug targets. The signalling pathways of these receptors proceed by engaging with four main types of G proteins (G_s_, G_i/o_, G_q_ and G_12_), which in turn result in different physiological responses. Investigating the mechanisms of the coupling between G proteins and receptors, and how selectivity is modulated, has become an important quest in the field to understand receptor pathophysiology and to inform better drug design.

Many high-resolution structures of G-protein-coupled receptors (GPCRs) have now been solved^1^ primarily by X-ray crystallography, NMR and more recently cryoelectron microscopy to gain insight into the architecture of receptors and the mechanism of their functionality. Mass spectrometry has also been used to understand the ability of different drug molecules or ligands to modulate the structural dynamics of GPCRs, including during the formation of complexes between G proteins and receptors^2–7^. The utility of mass spectrometry in monitoring ligand binding to GPCRs is compromised by the difficulties encountered in preserving these interactions in the gas phase. However, interactions between the purinergic receptor P_2_Y_1_ and its natural ligand ADP/ATP, or a synthetic ligand, were maintained in a mass spectrometer, while small-molecule ligands and peptide binding to the glucagon receptor were enhanced when high concentrations of sodium were present^8,9^.

The presence of small ligands such as lipids or ions (Na^+^, Ca^2+^ and Zn^2+^) in the structures of GPCRs has been reported, and their impact on the function and stability of various receptors has been proposed^10–13^. However, it remains challenging to interrogate receptor interactions directly and to illustrate the functional impact of these small molecules due to the absence of a methodology to simultaneously capture the effects of ligand binding and G-protein coupling. In this study we have developed and applied a native mass spectrometry (nMS) approach to interrogate the molecular pharmacology of a purified GPCR, the turkey β_1_-adrenergic receptor (tβ_1_AR). By capturing the G-protein-coupling activity of the receptor and monitoring its response to a range of agonists and antagonists, we delineated the propensity of receptor coupling to G_s_ and G_i_ modulated by different ligands. We demonstrate here the biased propensity of isoprenaline to stimulate G_i_-protein coupling and, unexpectedly, endogenous zinc ions, which preferentially stabilize complexes between G proteins and receptors. Following an in-depth investigation by means of nMS, molecular dynamics (MD) simulations and protein mutagenesis, we reveal a novel allosteric mechanism in which zinc ions coordinate the structural transition during the formation of complexes between G proteins and receptors, enabling us to propose a role for metal ions in modulating the selectivity of G-protein coupling.

## Results

### High levels of G-protein coupling are observed for agonist-bound tβ_1_AR

To develop our approach we selected tβ_1_AR as its molecular pharmacology is well understood. To facilitate protein purification and to retain functionality, we modified a previously described stabilized construct of tβ_1_AR (β44-m23^14,15^; see Methods). With the same truncation and deletion on the amino and carboxy termini and intracellular loop 3, two reversal mutations (A227Y and L282A) were introduced to enable full activation and high-affinity agonist binding of the receptor in the presence of G proteins and nanobodies^16,17^. The mutation R284K, equivalent to the residue of the β_2_-adrenergic receptor (β_2_AR), was included to improve the binding of the Nb6B9 nanobody^18^. In addition, the glutamic acid at position 130 was mutated to tryptophan (E130W) to allow purification of the receptor in its apo state by stabilizing the intrahelical interactions between transmembrane helices TM3, TM4 and TM5 (ref. ^19^). Cyclic AMP assays confirmed the activity of the engineered receptor in response to various ligands (Supplementary Fig. 1a).

**Fig. 1:**
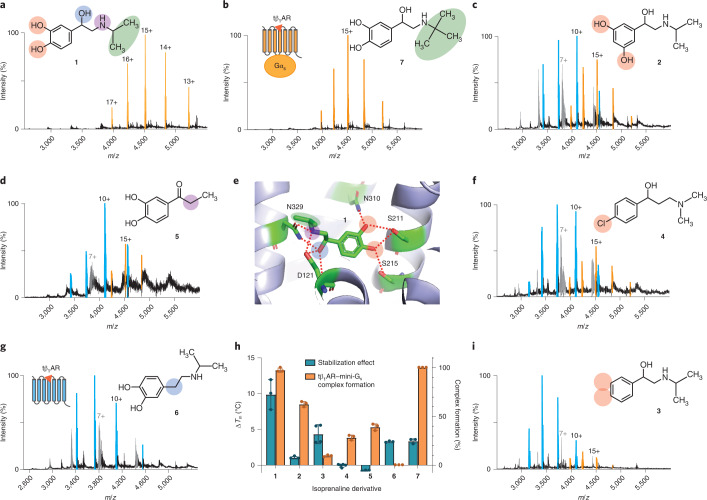
Interrogating the structure–function relationship of isoprenaline derivatives by nMS. **a**–**d**,**f**,**g**,**i**, Representative MS spectra of purified tβ_1_AR (5 μM) complexed with mini-G_s_ (6 μM) in the presence of various isoprenaline derivatives (each 250 μM): isoprenaline (**a**), colterol (**b**), orciprenaline (**c**), 3,4 dihydroxypropiophenone (**d**), 1-(4-chlorophenyl)-3-(dimethylamino)propan-1-one (**f**), isopropyldopamine (**g**) and 1-phenyl-2-(2-propylamino)ethan-1-ol (**i**). The peaks assigned to the receptor–mini-G_s_ complex, receptor monomer and mini-G_s_ are highlighted in orange, blue and grey, respectively, alongside the annotations of their charge states. The structures of the isoprenaline derivatives are illustrated and numbered alongside each spectrum. **e**, Structure of tβ_1_AR complexed with isoprenaline (PDB: 2Y03), showing the critical interactions between the receptor and isoprenaline, denoted by red dashed lines. **h**, Impact of isoprenaline derivatives **1**–**7** on the thermostability (Δ*T*_m_) and extent of complex formation (tβ_1_AR–mini-G_s_ complex/total tβ1AR) assessed by MS. Purified tβ_1_AR was pre-incubated with various isoprenaline derivatives **1**–**7** before the thermostability assay (see Methods), and the effect of different isoprenaline derivatives on the degree of stabilization was determined by the difference in the protein melting point (Δ*T*_m_) in the presence and absence of isoprenaline. The dots refer to the individual data points and the error bars in **h** represent the mean ± s.d. from at least three independent experiments. Source data

Mass spectrometry measurements of purified tβ_1_AR were optimized (see Methods). In addition to the exact mass of the protein, a modification of mass 132.8 ± 0.47 Da was apparent, assigned to *O*-xylosylation (Supplementary Fig. 1). To investigate the activity of the purified receptor, we next used mini-G_s_, an engineered Gα subunit that forms a stable complex with active receptors, to examine the extent of receptor coupling in the presence of a saturating concentration of the full agonist isoprenaline (**1**). We observed full complex formation (Fig. 1a), whereas previously only ~60% complex formation was detected for isoprenaline co-purified receptor without the E130W mutation^20^. Although the E130W mutation and isoprenaline treatment both substantially increased receptor stability, this result implies a better G-protein-coupling activity of this receptor, attributed to the stabilization of TM4–TM3–TM5 intrahelical interactions.

### Molecular pharmacology of tβ_1_AR is recapitulated by our nMS platform

To assess the sensitivity of our approach to changes in the structure of the agonist, we first investigated six derivatives of isoprenaline that have been well-characterized previously for their ability to attenuate the coupling between tβ_1_AR and G_s_ protein (Supplementary Fig. 2). The X-ray crystal structures of tβ_1_AR bound to isoprenaline highlight the main contacts with the receptor: the *meta- and para-*hydroxy groups of catecholamine form hydrogen bonds with Ser 211, Ser 215 and Asn 310, and the secondary amine and β-hydroxy group interact with Asp 121 and Asn 329 (Fig. 1e). We further examined the stimulatory activity of these compounds for the coupling between tβ_1_AR and G_s_ protein using our nMS platform. (It is important to note, however, that nMS analysis of membrane proteins is performed at elevated energies to remove bound detergents, which often leads to the removal of weakly bound ligands, which are then not observed in the mass spectra. Importantly, however, we capture the effect that ligand binding has in solution, and consequently on complex formation.) We first tested orciprenaline (**2**) and, then we tested 1-phenyl-2-(2-propylamino)ethan-1-ol (**3**) which abolished the interactions between the catecholamine aryl hydroxy groups and the receptor. The movement of the *para*-hydroxy group to the *meta* position 2 f resulted in an ~60% reduction of mini-G_s_-coupling (Fig. 1c). The removal of both aryl hydroxy groups in **3** led to 90% attenuation of coupling, aligning with previous observations that the two conserved serine residues on TM5 are crucial for agonism (Fig. 1i)^14^. Introduction of a chloride at para position in **4** led to a partial recovery of the coupling activity. Although the chloride was not expected to retrieve hydrogen bonding, its strong electronegativity may contribute to the formation of π-interactions between the phenyl ring of isoprenaline and adjacent serine residues of the receptor (Fig. 1f). Examining the impact of the secondary amine and β-hydroxy group with derivatives **5** and **6**, we found 75% attenuation and no coupling, respectively (Fig. 1d,g). Intriguingly, our mass spectrometry (MS) platform was able to detect the stimulatory activity of **4** and **5**, which were expected to have low binding affinity for the receptor, highlighting the sensitivity of the nMS platform.

Although MS is able to detect the downstream effects of these isoprenaline derivatives, it is challenging to observe direct binding under the detergent micelle conditions used here, as outlined above. To confirm the binding of these derivatives to the receptor, we therefore performed an orthogonal assay in which we assessed the increase in thermostability achieved following ligand binding to the receptor. We found that incubation of isoprenaline substantially improved receptor stability, with most of the other derivatives stabilizing the receptor to a lesser extent (Fig. 1h), showing that a certain degree of interactions between the derivatives and receptor were preserved. However, we did not observe any stabilization effect with two of the derivatives, 1-(4-chlorophenyl)-3-(dimethylamino)propan-1-one hydrochloride (**4**) and 3,4 dihydroxypropiophenone (**5**), although weak agonist activity was observed using nMS. Comparison across the ligand structures allowed us to deduce that the modifications in **6** retained binding competency but completely lost their stimulatory effect, whereas **5** remained active but compromised most of its binding to the receptor. Moreover, the trimethyl group in colterol (**7**) retained the same full-coupling activity as **1** (Fig. 1b).

**Fig. 2:**
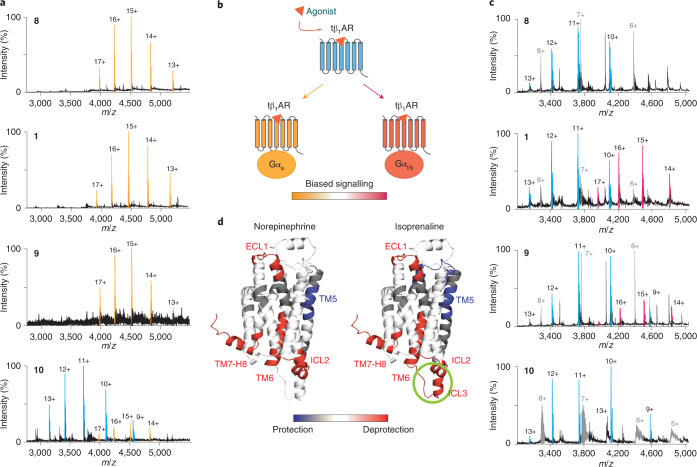
Characterizing the coupling of tβ_1_AR coupling to engineered Gα subunits. **a**, MS spectra of purified tβ_1_AR complexed with mini-G_s_ in the presence of the agonists isoprenaline (**1**), norepinephrine (**8**), carmoterol (**9**) and dobutamine (**10**) at a concentration of 25 μM. The peaks assigned to the receptor–mini-G_s_ complex and the receptor monomer are denoted in orange and blue, respectively. **b**, Schematic illustration of the stimulation propensity of agonists towards G_s_ and G_i_ proteins and the potential biased effect of agonists. **c**, Mass spectra of tβ_1_AR–mini-G_i/s_ complexes formed in response to agonists **1** and **8**–**10** at 25 μM. The signals of the tβ_1_AR–mini-G_i/s_ complex, receptor monomer and mini-G_s_ are shown in magenta, blue and grey, respectively. **d**, Different deuterium uptake upon tβ_1_AR activation in the absence of G proteins is induced by norepinephrine and isoprenaline and mapped onto the structure of tβ_1_AR (PDB: 2Y03). Increased uptake in comparison with the receptor without treatment is denoted in red, and decreased uptake is coloured blue. The ICL3 motif is uniquely modulated by isoprenaline, highlighted by the green circle. Representative spectra from three independent experiments are shown in **a** and **c**.

To further establish our nMS platform for molecular pharmacology characterization of full agonists (norepinephrine (**8**), carmoterol (**9**) and isoprenaline (**1**)), partial agonists (dobutamine (**10**) and salbutamol (**11))** and antagonists (cyanopindolol (**12**), carazolol (**13**) and carvedilol (**14**)), we added these well-characterized ligands individually to solutions containing the receptor and mini-G_s_. Complete complex formation was observed for the full agonists **8, 9** and **1** at saturation concentration, while the two partial agonists **10** and **11** elicited only a limited response under the same MS conditions (Fig. 2a and Supplementary Fig. 3). Little or no complex formation was detected for antagonists **12** and **13**, respectively (note that **12** is reported to be a weak agonist^21^). The sensitivity of MS detection to low populations of complexed receptor highlights our ability to provide a direct readout (Supplementary Fig. 3). Quantifying the extent of complex formation allowed us to plot dose–response curves for agonists and partial agonists, which revealed half-maximum effective concentration (EC_50_) values comparable to those reported previously, with the same rank order^22^. Addition of antagonist **13** led to the expected dose-dependent right shift of the isoprenaline curve, confirming the antagonistic action of **13** (Supplementary Fig. 3).

### Capturing biased signalling through G_i_-protein coupling

To explore the stimulatory propensity of drug molecules towards different G proteins, we carried out analogous experiments to those above with mini-G_i/s_ (the helix 5 (H5) motif of mini-G_s_ was replaced with the sequence of G_i_; Fig. 2b and Supplementary Fig. 4a). β-Adrenergic receptors have previously been reported to bind selectively to G_i_ proteins under certain stimulatory conditions^23,24^, suggesting a ‘switch’ mechanism to propagate different signalling events. Intriguingly, for the three full agonists **1, 8** and **9**, isoprenaline (**1**) induced mini-G_i/s_ coupling to a greater extent than **9**, whereas no complex formation was detected with the natural ligand **8** (Fig. 2c). The propensity of isoprenaline to stimulate G_i_-protein coupling of tβ_1_AR was further validated by a competition experiment in which the receptor was incubated with mini-G_s_ and mini-G_i/s_ at equimolar ratios. In line with the preferential G_s_ signalling of tβ_1_AR, substantially more coupling between the receptor and mini-G_s_ was observed than with mini-G_i/s_ in a competitive manner. Thus, derivative **1** was able to induce receptor complex formation with both mini-G_s_ or mini-G_i/s_, whereas the receptor–mini-G_i/s_ complex was not detected in the presence of the other full agonists **8** and **9** (Supplementary Fig. 4). These data are supported by the higher efficacy of isoprenaline compared with norepinephrine in triggering the dissociation of trimeric G_i_ proteins^25^, and indicate the propensity of isoprenaline to stimulate G_i_-protein coupling of tβ_1_AR.

To understand the structural changes associated with the isoprenaline-mediated biased effect, we performed hydrogen–deuterium exchange mass spectrometry (HDX-MS) on the receptor alone to investigate its conformational dynamics in the presence of the natural ligand norepinephrine (**8**) or isoprenaline (**1**). The overall differences in deuterium exchange of the receptor upon activation induced by these two agonists was very similar, with increased deuterium uptake in the motifs of extracellular loop 1 (ECL1), intracellular loop 2 (ICL2), the intracellular tip of TM6 and TM7–H8 (refs. ^17,26^; Fig. 2d and Supplementary Fig. 5). TM5, however, exhibited more protection in both cases following activation, consistent with a reduction in mobility upon ligand binding to the motif 2107218 of TM5. Moreover isoprenaline (**1**), but not norepinephrine (**8**), specifically promoted higher deuterium uptake in intracellular loop 3 (ICL3), consistent with increased conformational dynamics. We propose that the increased dynamics of this loop likely plays a role in promoting biased signalling pathways wherein alternative conformations might be facilitated.

### Endogenous zinc ions stabilize G_s_-protein coupling

**Fig. 3:**
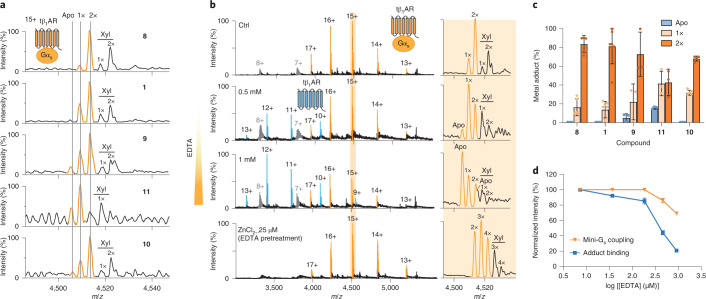
Identification of an endogenous metal in the tβ1AR–mini-G_s_ complex and its functional relation to G_s_-protein coupling. **a**, Endogenous metal adducts were detected in the tβ_1_AR–mini-G_s_ complex under stimulation with the agonists isoprenaline (**1**), norepinephrine (**8**), carmoterol (**9**), dobutamine (**10**) and salbutamol (**11**). The stoichiometry of metal binding is denoted above individual peaks (1×, one adduct; 2×, two adducts). **b**, The impact of EDTA on tβ_1_AR–mini-G_s_ complex formation and its association with the endogenous metal. The binding stoichiometry of the metal ion is indicated 1×–4×, denoting one to four adducts. A supplement of exogenous ZnCl_2_ at 25 μM added to EDTA-pretreated receptor and mini-G_s_ led to the recovery of complex formation of tβ_1_AR and mini-G_s_ (bottom spectrum). The peaks assigned to the receptor–mini-G_s_ complex, receptor monomer and mini-G_s_ are highlighted in orange, blue and grey, respectively**. c**, Percentage of endogenous metal adduct with different stoichiometries normalized to the total intensity of the tβ_1_AR–mini-G_s_ complex in response to different tβ_1_AR agonists. The dots refer to the individual data points and the bars represent the mean ± s.d. from a variable number of independent experiments (*n* = 5 for norepinephrine (**8**) and dobutamine (**10**); *n* = 7 for isoprenaline (**1**); *n* = 3 for carmoterol (**9**) and salbutamol (**11**)). **d**, Quantification of tβ_1_AR–mini-G_s_ complex formation and its association with metal ion in the presence of EDTA at different concentrations. The relative percentage was calculated by normalizing to the condition without EDTA. The data points represent the mean ± s.d. from three independent experiments. Source data

While investigating the formation of complexes between tβ_1_AR and mini-G proteins, we observed an additional peak with an added mass of 64.2 ± 1.4 Da present in all receptor–mini-G_s_ complexes. The binding stoichiometry of this additional peak (1 or 2), which we tentatively assigned to zinc or copper ions based on mass, was found to vary with different agonists. For the natural ligand norepinephrine (**8**), the predominant complex contained two metal ions. The absence of a statistical distribution of ligand-bound species implies specific ion binding and a potential role for this endogenous ion in modulating G-protein coupling (Fig. 3a,c). Given the measured mass of this ion, tentatively identified as zinc or copper ions, we treated the receptor complex solution with EDTA to chelate divalent cations and monitored the impact of this chelation on G-protein coupling (Fig. 3b). A substantial reduction in the level of receptor in the complex with the adduct was observed together with lower levels of the receptor–mini-G_s_ protein complex (Fig. 3b,d). We anticipated that the adduct would be Zn^2+^ ions, given their observed effect in a previous cell-based study^27^, although the molecular details were not ascertained. To confirm the presence of Zn^2+^ ions, we conducted inductively coupled plasma mass spectrometry (ICP-MS) to examine the trace elements in our protein solutions. Intriguingly, a very low amount of zinc (<5 ppb) was detected in the purified receptor solution, but no zinc ions were detected in the buffer solutions. Divalent zinc ions must therefore be endogenously sourced from the cell lines used for receptor overexpression (Supplementary Fig. 6). Given the minute amount of zinc ions, this result highlights the sensitivity of nMS for detecting direct interactions between the receptor and Zn^2+^ ions. Moreover, the addition of exogenous zinc ions to EDTA-pretreated receptor and mini-G_s_ led to full recovery of the receptor–mini-G_s_ complex (Fig. 3b). High levels of Zn^2+^ led to the formation of multiple zinc ion adducts that are likely to be the result of non-specific binding. Importantly, the addition of copper ions did not alter receptor–mini-G_s_ complex formation substantially (Supplementary Fig. 7). Moreover, reducing the concentration of the agonists isoprenaline and carmoterol (fivefold less than the receptor; Supplementary Fig. 3) led to a substantial reduction in the population of the receptor–mini-G_s_ complex, but the complex maintained the preferential binding to zinc ions. Together these experiments align with our hypothesis that zinc ions serve as an essential allosteric modulator for G-protein coupling, even at lower (not saturating) ligand concentrations.

**Fig. 4:**
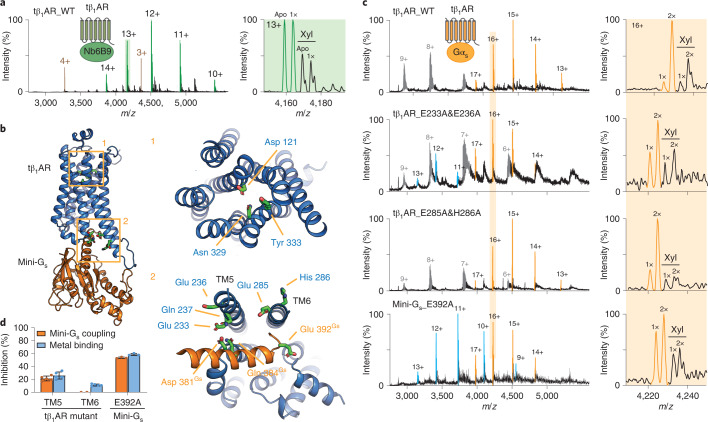
Localization of zinc-binding hotspots and their functional impact on G_s_-protein coupling. **a**, MS spectrum of the tβ_1_AR–Nb6B9 complex incubated with isoprenaline, indicates reduced binding of endogenous metal. The complex is highlighted in green and its metal-binding stoichiometry is reduced to first order (denoted as 1×). The peaks assigned to Nb6B9 alone are highlighted in brown. **b**, MD simulation of the tβ_1_AR–mini-G_s_ complex in the presence of zinc revealed two zinc-binding hotspots. The zinc contacts in the orthosteric ligand-binding site (Asp 121, Asn 329 and Tyr 333) are shown in the upper box (labelled 1), and a subset of the zinc contacts at the intracellular interface are highlighted on the receptor (Glu 233, Glu 236, Gln 237, Glu 285 and His 286) and mini-G_s_ (Asp 381, Gln 384 and Glu 392) in the bottom box (labelled 2). **c**, The impact of mutations to the zinc contacts on tβ_1_AR–mini-G_s_ complex formation and metal binding (orange boxes). Two receptor variants (E233A&E236A and E285A&H286A) and a mini-G_s_ mutant (E392A) were examined. Representative MS spectra from three independent experiments are shown. The receptor monomer and receptor–mini-G_s_ complex are shown in blue and orange, respectively. **d**, The inhibitory effect of zinc-contact mutants on tβ_1_AR–mini-G_s_ complex formation (orange bar) and metal binding (blue bar). The percentage inhibition was calculated from the results acquired with wild-type tβ_1_AR and mini-G_s_, shown in the top spectrum of **c**. The dots represent the individual data points and the bars represent the mean ± s.d. from three independent experiments on the same batch of purified proteins. Source data

To gain structural insights into the possible interactions of zinc with tβ_1_AR, we carried out MD simulations at atomic resolution for tβ_1_AR, in inactive or active conformations, in a lipid bilayer with Zn^2+^ ions present in the aqueous phase (Supplementary Fig. 8). The simulations revealed dynamic zinc interactions with the receptor in two putative areas on the extracellular motifs: one surrounding the orthosteric ligand-binding site and the other corresponding to the cytoplasmic interface of the TM3, TM5 and TM6 helices. Given that zinc ions interact with the receptor cytoplasmic interface, where conformational movement plays a critical role in G-protein coupling, we next examined Zn^2+^ binding with the tβ_1_AR–mini-G_s_ complex (Fig. 4b). Overall, the Zn^2+^ binding contacts are similar to those of the receptor alone: residues Asp 121, Asn 329 and Tyr 333 from the orthosteric pocket and Glu 233, Glu 236, Gln 237, Glu 285 and His 286 from the cytoplasmic interface form interactions with zinc. Intriguingly, we observed that residues of mini-G_s_, Asp 381, Gln 384 and Glu 392 on the H5 helix and Asp 354 on the α4–β6 loop, also contribute to binding, suggesting that the complex forms more stable contacts with Zn^2+^ (Supplementary Fig. 8), implying the potential role of Zn^2+^ in stabilizing G-protein coupling.

To probe the possible involvement of the Zn^2+^ binding sites observed in β_1_AR function, we used a nanobody (Nb6B9) that specifically recognizes the active receptor. Given that the structures of receptors bound to Nb6B9 or to mini-G_s_ are virtually identical (root mean squared displacement = 0.4–0.6 Å)^18,28^, the use of Nb6B9 provides an appropriate reference for investigating the impact of G-protein coupling on zinc binding. Compared with the complex between the G protein and receptor, the mass spectrum of the tβ_1_AR–Nb6B9 complex, following stimulation with isoprenaline, revealed substantially less Zn^2+^ binding (Fig. 4a). This observation indicates the presence of a G-protein-specific zinc binding site at the intracellular interface between the receptor and G protein.

To further interrogate the contact residues that are important for the effect of Zn^2+^ on G-protein coupling, we generated two tβ_1_AR variants with alanine mutations on the TM5 (E233A and E236A) and TM6 (E285A and H286A) motifs. The results reveal that the TM5 mutant attenuates endogenous zinc binding and coupling activity by 25.8 ± 5.4 and 21.2 ± 3.8%, respectively, whereas the TM6 mutant shows only a slight reduction (11 ± 1.56%) of endogenous zinc binding (Fig. 4c,d). Our results are distinct from previously published data that revealed that the mutation of Glu 225 of β_2_AR (equivalent residue to Glu 233) did not abolish the allosteric effect of zinc on the cAMP response observed in cell-based assays^27^. To exclude the potential adverse effect of mutations on receptor conformation, we examined the binding activity of Nb6B9 towards each mutant. We found that the mutations had no effect on agonist-induced receptor–nanobody complex formation (Supplementary Fig. 9). Our results imply, therefore, that the attenuated activity observed in the TM5 mutant is G_s_-protein-specific and likely caused by reduced Zn^2+^ binding.

We next explored the binding contact with mini-G_s_ by introducing an alanine mutation into mini-G_s_ at Glu 392. This point mutation resulted in a substantial decrease in both endogenous Zn^2+^ and the interactions between receptor and G_s_ protein by 58.3 ± 1.57 and 53.1 ± 1.1%, respectively (Fig. 4c,d). We further performed a potential mean force (PMF) calculation to examine the effect of zinc binding on the free-energy landscape of interactions between tβ_1_AR and mini-G_s_. The calculation indicated that zinc binding at the interface of the complex stabilizes the association by ~15 kJ mol^−1^ relative to the apo state (Supplementary Fig. 9). Collectively, our results suggest that the endogenous zinc ion functions as a positive allosteric modulator of G_s_-protein coupling through the stabilization of the interface between the receptor and G_s_ protein.

### Endogenous zinc facilitates the structural transition of G_s_-protein coupling

**Fig. 5:**
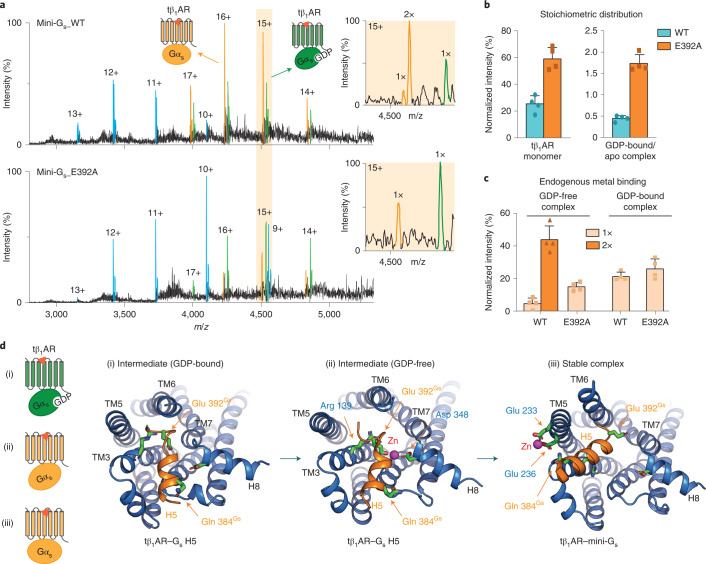
The allosteric effect of endogenous zinc on the selectivity of mini-G_s_. **a**, Representative MS spectra of tβ_1_AR coupling to wild-type (WT) mini-G_s_ and the E392A mutant in response to isoprenaline at 25 μM. The GDP-bound and GDP-free complexes are highlighted in green and orange, respectively, and the receptor monomer is indicated in blue. The binding stoichiometry of the metal ion is shown in the orange boxes. **b**, Relative percentages of the stoichiometric distribution of the tβ_1_AR monomer and tβ_1_AR–mini-G_s_ GDP-bound and GDP-free complexes. The results indicate weaker complex formation and slower structural transition to the stable state in mini-G_s_ E392A. **c**, Binding stoichiometry of the metal ion in the intermediate and stable complexes of tβ_1_AR–mini-G_s__WT and tβ_1_AR–mini-G_s__E392A. The bars in **b** and **c** show the mean ± s.d. from three independent experiments, including individual data points, on the same batch of purified proteins. **d**, Schematic representation of the hypothesized activation mechanism of tβ_1_AR–G_s_ complex formation according to molecular dynamic simulations performed on the tβ_1_AR–G_s_ H5 peptide and tβ_1_AR–mini-G_s_ complexes. The receptor first forms contacts with the G_s_ protein in a GDP-bound state (i), as an intermediate complex it further transits to a conformation where zinc interacts with both Glu 392^Gs^ and Asp 348^β1AR^ (ii), leading to the formation of a stable complex in a GDP-free state (iii). Snapshots of the MD structures illustrate the spatial orientation of the H5 motif of the G_s_ protein (orange) and its coordination with Zn^2+^ in the discrete conformations of the complex. Source data

The potential involvement of Gα_s_-specific residues Gln 384 and Glu 392 in Zn^2+^ binding, and our observation of reduced zinc binding in the tβ_1_AR–mini-G_i/s_ complex (Supplementary Fig. 4d), suggest that endogenous zinc might modulate the selectivity of G proteins. A structure reported for β_2_AR complexed with the Gα_s_ H5 motif addressed the role of Gα_s_ Glu 392 in the formation of the receptor–G_s_ complex in the guanosine diphosphate (GDP)-bound state^29^. That study presented an intermediate configuration of complex formation, where Arg 389 and Glu 392 in Gα_s_ contact Thr 68, Arg 131 and Tyr 141 of β_2_AR. To investigate the potential for Zn^2+^ to influence this intermediate complex stage, and thereby the selectivity of G_s_-protein coupling, we performed MS experiments with mini-G_s_ coupling to tβ_1_AR with very short incubation times (*t* < 1 min). The tβ_1_AR–mini-G_s_ complex was detected immediately after adding isoprenaline, indicating the high rate of complex formation. Intriguingly, in addition to the GDP-free complex, we observed the presence of the GDP-bound complex, aligning with the hypothesis of intermediate complex formation (Fig. 5a). We next examined the coupling of mini-G_s_ with the E392A variant under the same experimental conditions, again with very short incubation times (*t* < 1 min), and observed a 35% reduction in complex formation in comparison with the wild-type mini-G_s_. Furthermore, the intensity ratio of the GDP-bound and GDP-free complexes was substantially higher for the E392A mutant, indicating a lower rate of transition from the intermediate to the stable state (Fig. 5a,b).

It is of interest that the extent of endogenous zinc binding observed in the mass spectra was dramatically reduced in the complex between the mini-G_s_ E392 mutant and tβ_1_AR following short incubation times compared with longer incubation times (83.1% versus 53.1%). By contrast, no substantial difference was observed between the mutant and wild-type metal ion binding in the GDP-bound state (Fig. 5a,c). We infer, therefore, that G_s_ Glu 392 is not involved in zinc binding at the GDP-bound complex stage, before structural rearrangement of the nucleotide-binding pocket of G_s_.

To further investigate the role of Glu 392 in complex formation, we carried out atomic simulations for the tβ_1_AR–G_s_ H5 complex in the intermediate configuration and identified a further conformational state where a zinc ion binds to Glu 392 of G_s_ H5 and Asp 348 of the receptor. The position of this zinc ion suggests that it induces an upward rotation of G_s_ H5, which could be important structurally for the transition of the tβ_1_AR–G_s_ intermediate complex to the stable state (Fig. 5d), as seen in the analogous complex of β_2_AR with trimeric G_s_. In addition to the Glu 392 mutation in mini-G_s_, we examined the coupling activity of the tβ_1_AR D348A mutant and its association with endogenous metal (Supplementary Fig. 10). The mutation of Asp 348 at position 8.49 (Ballesteros–Weinstein numbering scheme) unexpectedly increased the extent of complex formation with mini-G_s_. This observation coincides with previous studies that showed that the ionic interactions between negatively charged glutamic acid (E^8.49^) and positive residue arginine (R^2.46^) are important for stabilizing the receptor in an inactive conformation^30,31^. The structure of inactive tβ_1_AR (PDB: 2VT4) also presents a similar ionic lock between Asp 348 and Arg 71; hence the mutation of Asp 348 is suggested to destabilize the inactive receptor, in line with our results. Although it is not possible to use this variant to validate the role of Asp 384 in the structural transition of the intermediate complex, the binding of endogenous metal to tβ_1_AR D348A is substantially increased in its monomeric state, highlighting the functional correlation of metal binding to receptor activity (Supplementary Fig. 10).

## Discussion

Here we have demonstrated a novel MS approach to investigating the pharmacology of GPCRs. Combining the sensitivity and near-atomic resolution of the mass spectrometer, while preserving the interactions between receptor and G protein, our methodology is capable of monitoring, with very high sensitivity, the full spectrum of receptor pharmacology in vitro. This has led to the discovery of endogenous zinc as an allosteric modulator of receptor activation. Overall, our results allow us to propose that the fine-tuning of agonists, antagonists and allosteric modulators can be captured on one platform with the results interpreted with the aid of MD simulations, HDX-MS, site-directed mutagenesis and the plethora of high-resolution structures of GPCRs, and that endogenous zinc facilitates the structural transition of the receptor–G_s_ complex from the intermediate (GDP-bound) to the stable (GDP-free) state. Given that the interaction of G_s_ Glu 392 with zinc triggers the upward rotation of the H5 motif, and that Glu 392 is largely exposed to solvent in the stable complex, with no specific interaction observed with the receptor (Supplementary Fig. 10), its interaction with endogenous Zn^2+^ may play a critical role in modulating the selectivity of G_s_.

An understanding of GPCR agonism and allostery has been emerging in recent years^32^. The conformational plasticity of receptors reflects the versatility of ligands in modulating receptor activity. Multiple allosteric sites, ranging from the extracellular and transmembrane domains to the cytoplasmic face, have been described for various receptors^33^. However, it is challenging to predict receptor allostery due to an absence of conserved allosteric sites and mechanistic insight into their action. The MS approach presented here to interrogate receptor pharmacology in high definition and the discovery of endogenous Zn^2+^ ions as an allosteric modulator exemplify the utility of this platform. Moreover, the involvement of the H5 motif of the Gα subunit in allosteric regulation highlights the role of the G protein itself in receptor allostery. A similar phenomenon was observed previously for a specific lipid, phosphatidylinositol-4,5-bisphosphate, which improves the activity of coupling by bridging between the receptor and the G protein^20^. The data presented here offer an enhanced understanding of the regulation of the interaction between receptor and G protein. Given that zinc coordinates with the selectivity determinant of G_s_, the potential to regulate its binding or to mimic its mechanism by high-potency small molecules may provide a new avenue for modulating the kinetics of the specific signalling of receptor–G protein for therapeutic purposes. Overall, our study offers a novel perspective for the rational design of GPCR agonists and allosteric modulators.

## Methods

### Constructs and proteins

In our experiments we used the expression plasmid of a thermostabilized variant of turkey (*Meleagris gallopavo*) β_1_AR. The synthesized cDNA encoding tβ_1_AR, flanked with N-terminal Flag and Strep tags and C-terminal His tag, was cloned into the pFastBac1 vector via NheI and NotI cloning sites. The protein sequence ranged from residues 44–364 with ICL3 truncation (244–272) and contained seven thermostabilizing point mutations (R68S, C116L, E130W, D322K, F327A, F338M and C358A). Purified β_1_AR, engineered Gα_s_ (mini-G_s_) and nanobody Nb6B9 were used for mass spectrometry analysis.

### Expression and purification of mini-G_s_ and mini-G_i/s_

The engineered minimal G protein, mini-G_s_ construct R414 and mini-G_i/s_ construct R43 cloned into the pET24a vector were expressed in *Escherichia* *coli* BL21(DE3) strain and purified by Ni^2+^ affinity chromatography, followed by cleavage of the histidine tag using tobacco etch virus (TEV) protease. The cleaved tag, protease and undigested mini-G proteins were removed by reverse immobilized metal affinity chromatography (IMAC) purification on Ni^2+^-nitrilotriacetic acid (NTA). Proteins were concentrated to 2 mg ml^–1^ in 20 mM HEPES (pH 7.5), 100 mM NaCl, 10% v/v glycerol, 1 mM MgCl_2_ and 10 mM GDP.

### Expression and purification of tβ_1_AR

The construct of tβ_1_AR was overexpressed in insect cells using the Bac-to-Bac baculovirus expression system (Thermo Fisher). The recombinant baculoviruses prepared using the expression vector pFastBac1 (Thermo Fisher) were used to infect Sf9 cells (Invitrogen, 11496015) with the multiplicity of infection between 1 and 2. The cell membrane was enriched and solubilized in 20 mM Tris-HCl (pH 8), 350 mM NaCl, 3 mM imidazole and 1.5% w/v *n*-dodecyl-β-d-maltopyranoside (DDM; Anatrace) for 15 min. The supernatant was isolated by ultracentrifugation at 175,000 *g* for 1 h and loaded onto a HiTrap TALON crude column (GE Healthcare) for affinity enrichment. The column was washed with ten column volumes of 20 mM Tris-HCl (pH 8), 350 mM NaCl, 3 mM imidazole and 0.05% DDM after loading the supernatant, and the receptor was eluted with a gradient of 20 mM Tris-HCl (pH 8), 350 mM NaCl, 250 mM imidazole and 0.05% DDM in three column volumes. The pH of all the buffers was adjusted at room temperature. The fractions containing receptor were pooled and concentrated to a final concentration of 2–3 mg ml^–1^ using an Amicon centrifugal filter with a molecular weight cut-off of 50 kDa for subsequent application. To mitigate experimental variations that may cause differential binding with endogenous zinc ions, we carefully controlled our experimental conditions during the purification of the various tβ_1_AR mutants. Specifically, the quantity of starting biomass (20 g), ratio of cell membrane and detergent (DDM/membrane protein = 3:1, w/w), duration of detergent solubilization (15 min) and fast protein liquid chromatography (FPLC) conditions were strictly controlled.

### Expression and purification of nanobody Nb6B9

The expression gene of Nb6B9 was cloned into the plasmid pET-26b(+) (ref. ^34^) containing a N-terminal His-tag followed by a thrombin protease cleavage site. Protein was overexpressed in *E. coli* BL21(DE3) strain (Agilent) and purified from the periplasmic fraction by Ni^2+^ affinity chromatography. The His-tag was removed with the use of a thrombin protease (Sigma) before concentration to 20 mg ml^–1^.

### Non-denatured mass spectrometry of tβ_1_AR

Purified β_1_AR was buffer-exchanged into 200 mM ammonium acetate buffer (pH 7.4) containing the mixed micelle preparation (DDM/Foscholine16/ cholesteryl hemisuccinate (CHS) = 20:2:3, w/w/w) optimized for GPCR analysis, as described previously^8^, before MS analysis using a modified Q-Exactive mass spectrometer (Thermo Fisher Scientific)^35^. A capillary voltage (1.1 kV) was applied during nanoelectrospray, and an optimized acceleration voltage (120 V) was then applied to the higher-energy C-trap dissociation (HCD) cell to remove the detergent micelle from the protein ions, following a gentle voltage gradient (injection flatapole, interflatapole lens, bent flatapole and transfer multipole: 7.9, 6.94, 5.9 and 4 V, respectively). To analyse receptor–mini-G_s_ complex formation, an optimized voltage was applied to the in-source fragmentation (100 V) and HCD cell (100 V) with the same voltage gradient for ion transmission. Spectra were acquired and averaged with a noise level parameter of 3. The back pressure was maintained at ~0.9 × 10^−9^ mbar. Data were analysed using Xcalibur 2.2 and the relative percentage of tβ_1_AR in different binding stoichiometries was quantified using the UniDec software^36^. Errors in measurement were derived from the deviation of peak centroids of differently charged states of the same mass species.

### Mini-Gα and Nb6B9 coupling to tβ_1_AR

Effector coupling to tβ_1_AR was analysed using a modified Q-Exactive mass spectrometer after incubating purified tβ_1_AR with mini-G_s_ or Nb6B9 in a 1:1.2 molar ratio at 4 °C in the coupling buffer (10 mM HEPES, 10 mM Tris-HCl (pH 7.4), 200 mM NaCl, 1 mM MgCl_2_, 5 mM GDP and 0.05% DDM) containing 25 μM agonist for at least 20 min. To strip the exogenous metal ligand, both purified tβ_1_AR and mini-G_s_ were pretreated with 5 mM EDTA for 5 min at 4 °C and then buffer-exchanged into EDTA-free buffers for tβ_1_AR (20 mM Tris-HCl (pH 8), 350 mM NaCl and 0.05% DDM) and mini-G_s_ (20 mM HEPES (pH 7.5), 100 mM NaCl, 1 mM MgCl_2_ and 10 mM GDP), respectively. The relative percentage of effector coupling was quantified using UniDec software, and the degree of effector coupling was calculated by normalizing the relative percentage of the complex to the sum of the percentage of receptor monomer and complex. To examine the inhibitory effect of antagonist, purified tβ_1_AR was pre-incubated with carazolol at the desired concentration for 10 min at 4 °C, followed by the same procedure described above for mini-G_s_ coupling in the presence of isoprenaline. All data analysis was carried out using GraphPad Prism 7 (GraphPad).

To investigate the intermediate complex formation, purified tβ_1_AR and mini-G_s_ were buffer-exchanged into 200 mM ammonium acetate buffer (pH 7.4) containing the mixed micelle preparation and 5 mM GDP. tβ_1_AR was premixed with mini-Gα in a molar ratio of 1:1 at 4 °C and the protein mixture was introduced into the mass spectrometer immediately after adding isoprenaline to a final concentration 25 mM. Spectra were acquired for 1 min and the relative percentages of tβ_1_AR monomer, tβ_1_AR–mini-G_s_ intermediate and stable complex were quantified using the UniDec software.

### Nano differential scanning fluorimetry for measuring the stability of purified tβ_1_AR

The stability of purified tβ_1_AR was meansured by nano differential scanning fluorimetry (NanoDSF). tβ_1_AR was diluted to 0.4 mg ml^–1^ in protein buffer (20 mM Tris-HCl (pH 8), 0.35 M NaCl, 3 mM imidazole and 0.05% DDM). β_1_AR ligands such as agonists, agonist derivatives, antagonists and partial agonists were tested at concentrations of 100 mM to measure their impact on receptor stability. Dimethylsulfoxide was added to a concentration of 5% in the final reaction volume. Control experiments involving protein alone, protein in 5% dimethylsulfoxide and ligands alone were conducted as references for measuring the stabilization effect of ligands. The protein sample and ligand were mixed at a fixed molar ratio (receptor/ligand, 1:10) and incubated for 20 min on ice before loading into NT.Plex nanoDSF-grade capillaries (NanoTemper). The melting curves of tβ_1_AR were determined using Prometheus Melting Control v1.9 software (NanoTemper) by measuring the intrinsic protein fluorescence signal and its change during a temperature ramp from 20 to 95 °C at a rate of 2 °C min^–1^. The melting temperature of the receptor was measured in triplicate and an average melting temperature was obtained.

### HDX-MS of purified tβ_1_AR

The equilibration buffer (E) was composed of 20 mM Tris-HCl (pH 8), 0.35 M NaCl, 3 mM imidazole and 0.05% DDM. The quench buffer (Q) was composed of 50 mM K_2_HPO_4_, 50 mM KH_2_PO_4_, 0.1% DDM and 100 mM tris(2-carboxyethyl)phosphine hydrochloride (TCEP. HCl). The labelling buffer (L) had the same composition as buffer E except H_2_O was replaced with D_2_O (99.8%). For drug treatment, 300 μM of drug was pre-incubated with the protein samples before deuterium labelling. Deuterium labelling was performed by diluting 5 μl of protein at a concentration of 16 μM in 95 μl of buffer L. The protein sample was incubated for various times and then quenched with buffer Q at 1 °C and pH 2.3. Samples were immediately digested with a pepsin column connected to an high-performance liquid chromatography (HPLC) system. For peptide analysis, the HPLC run time was 11 min at a flow rate of 40 μl min^–1^ under a gradient between buffer A (0.1% formic acid in H_2_O) and buffer B (acetonitrile with 0.1% formic acid). A C18 trap column (ACQUITY UPLC BEH, 1.7 μm, Waters) and C18 column (ACQUITY UPLC BEH, 1.7 μm, 1.0 mm × 100 mm, Waters) were used during the experiments. MS analysis was conducted in the range *m*/*z* = 100–2,000 in positive ion mode on a Synapt G2-Si mass spectrometer with an electrospray ionisation (ESI) source and ion mobility cell, coupled to an ACQUITY UPLC instrument with HDX automation technology (Waters). HDX analysis was performed at four time points (15 s, 2, 30 and 120 min). A buffer blank solution was injected between each analytical injection to remove any carryover. Data were obtained in triplicate at each time point, and were processed and analysed using MassLynx v4.1 (Waters). ProteinLynx Global Server (PLGS) software was used to analyse the MS data of unlabelled peptides and generate peptide libraries for each target protein. DynamX 3.0 (Waters) was used to analyse and quantify the deuteration of each peptide, and Deuteros 2.0 was used to assess statistically significant differences in deuterium uptake for peptides in two different conditions. The HDX data for each ligand bound to tβ_1_AR were mapped onto the published structure (PDB: 2YCW).

### ICP-MS analysis

tβ_1_AR, mini-G_s_ and their respective buffers were digested in digest solution on a hotplate using 0.3 M HNO_3_. The samples were analysed for trace elements using a PerkinElmer NexION 350D quadrupole inductively coupled plasma (ICP) mass spectrometer. Each element was calibrated against a series of calibration standards, which were robotically prepared with an Elemental Scientific prepFAST M5 autosampler. The stock standards were freshly prepared from a collection of synthetic ICP elemental standards (Merck Certipur, single element and custom blend) and diluted into 2% v/v HNO_3_. The ICP mass spectrometer was set up to measure a selection of elements together in one single method using the PerkinElmer Syngistix ICP-MS software. The ICP mass spectrometer was also equipped with a dynamic reaction/collision cell to suppress molecular interference and improve detection and accuracy.

### cAMP accumulation assay

Chinese hamster ovary (CHO) cells from Merck (85051005), maintained in DMEM/F12 cell culture medium supplemented with 10% fetal bovine serum and 1% l-glutamine, were grown to 70–80% confluence before transfection of engineered tβ_1_AR using FuGENE HD reagent (Promega) according to the manufacturer’s instructions. The next day, CHO cells transiently expressing engineered tβ_1_AR were prepared as a cell suspension in assay buffer (Hank’s balanced salt solution (HBSS) containing 5 mM HEPES (pH 7.4), further supplemented with 0.1% w/v BSA and 500 mM 3-isobutyl-1-methylxanthine) before being incubated with a range of concentrations of β-adrenoceptor ligands (noradrenaline, isoprenaline, carmoterol, dobutamine, salbutamol, cyanopindolol and carazolol), a negative control (assay buffer) and a positive control (10 mM isoprenaline and 3 mM forskolin) for 1 h at room temperature. After 1 h, cAMP levels were measured using the homogeneous time resolved fluorescence (HTRF) cAMP G_s_ HiRange kit (CisBio) according to the manufacturer’s instructions with fluorescence resonance energy transfer (FRET) levels being detected on a PHERAstar plate reader (BMG) equipped with BMG Reader Control v5.7 software; FRET ratios were calculated using the BMG MARS v4.01 software. All other data were analysed using GraphPad Prism 8 (GraphPad), including the conversion of FRET ratios to cAMP levels using a cAMP standard curve constructed in the same experiment.

### MD simulations

The coordinates of the inactive and active states of tβ_1_AR were taken from PDB structures 4BVN and 6H7N, respectively. The coordinates of the active tβ_1_AR complexed with mini-G_s_ were constructed by combining those of the active tβ_1_AR (PDB: 6H7N) with those of the mini-G_s_ in the adenosine A_2A_ receptor (A_2A_R–mini-G_s_ complex (PDB: 5G53) by aligning β_1_AR with A_2A_R. The missing ICL3 was stabilized by connecting the end residues Asp 242 and Ser 273. The protein structures were placed in a 10 × 10 nm^2^ membrane containing 80% POPC and 20% CHOL using CHARMM-GUI^37^, and then solvated with TIP4P water molecules at margins of 1.5 nm from the proteins. The systems were then neutralized with 150 mM NaCl and 0.35 mM ZnCl_2_. Three replicas were constructed for each conformational state with different membrane configurations. The MD simulations were performed using the GROMACS 2018 package^38^ and the CHARMM 36 force field for proteins^39^ and lipids^40^. The LINCS^41^ method was used to restrain all bonds, allowing for a save integration of 2 fs. Lennard-Jones and Coulomb cut-off distances were set to 1.2 nm, and the neighbour search cut-off was set to 1.2 nm with an update frequency of 10 fs. The particle mesh Ewald method was used to treat long-range electrostatic interactions.

The starting configurations were subjected to steepest descent minimization to remove close contacts. The systems were then slowly heated to 303 K using an NVT ensemble with a V-rescale thermostat. After that, each system was equilibrated for 10 ns using an NPT ensemble in which the pressure was kept constant at 1 bar by semi-isotropic coupling to a Parrinello–Rahman barostat with relaxation times (*τ*_P_ ) = 5.0 ps and a compressibility of 4.6 × 10^−5^ bar, and the temperature was maintained at 303 K by coupling (*t*_T_ = 0.5 ps) the protein membrane and solvent to a Nosé–Hoover thermostat. Throughout the heating and equilibration process, a harmonic position restraint was added to the protein backbone atoms and lipid head groups. The production run used the same parameters as the equilibration step, except for the positional restraints. In total, 500 ns of simulation data were collected from each simulation replica.

The Zn^2+^ binding sites were calculated from the simulation data using PyLipID (github.com/wlsong/PyLipID). The binding sites were identified from the community structures of the network, that is, groups of nodes that are more densely connected internally than with the rest of the network. The Zn^2+^ binding sites were determined from the simulations of inactive β_1_AR, active β_1_AR and active β_1_AR complexed with mini-G_s_. The residence times of Zn^2+^ in the binding sites showed a prominent increase in the last two simulations compared with in the simulations of the inactive β_1_AR.

To study the effect of Zn^2+^ on the association between β_1_AR and mini-G_s_, we calculated the PMF of mini-G_s_ dissociation from β_1_AR in the presence and absence of ZnCl_2_. The final system snapshot was taken from one replica of β_1_AR–mini-G_s_ simulations. To calculate the PMF in the absence of ZnCl_2_, zinc and chloride ions were removed from the systems and then the systems were further equilibrated. To generate configurations for umbrella sampling, steered MD was carried out to pull mini-G_s_ away from β_1_AR along the *z* axis (perpendicular to the membrane plane). The distance between the centre of mass of β_1_AR and the H5 motif of mini-G_s_ was monitored to ensure a pulling speed of 0.1 nm ns^–1^ with a force constant of 1,000 kJ mol^–1^ nm^–2^). The starting configurations for the umbrella sampling were extracted from SMD trajectories with a spacing of 0.1 nm along the monitored distance; 35 windows were generated, and each window collected 300 ns of simulation data. The PMF was extracted from the umbrella sampling using the weighted histogram analysis method (WHAM) provided by the GROMACS *g_wham* tool. A Bayesian bootstrap was used to estimate the statistical error of the energy profile.

### Reporting summary

Further information on research design is available in the Nature Research Reporting Summary linked to this article.

## Online content

Any methods, additional references, Nature Research reporting summaries, source data, extended data, supplementary information, acknowledgements, peer review information; details of author contributions and competing interests; and statements of data and code availability are available at 10.1038/s41557-022-01041-9.

### Supplementary information

Supplementary Information Supplementary Figs. 1–10.

Reporting Summary

Supplementary Data 1 Statistical data for Supplementary Fig. 1a.

Supplementary Data 2 Statistical data for Supplementary Fig. 4b.

Supplementary Data 3 Statistical data for Supplementary Fig. 6.

Supplementary Data 4 Statistical data for Supplementary Fig. 9d.

## Data Availability

The data supporting the main conclusions of this study are presented in the Article and Supporting Information. Additional data that support the findings of this study are available via Figshare as follows: nMS data are available at 10.6084/m9.figshare.19688640 and HDX-MS data are available at 10.6084/m9.figshare.19754662.v1. Source data are provided with this paper.

## Source data

Source Data Fig. 1 Statistical source data.
Source Data Fig. 3 Statistical source data.
Source Data Fig. 4 Statistical source data.
Source Data Fig. 5 Statistical source data.
