# Mass spectrometry captures biased signaling and allosteric modulation of a G protein-coupled receptor

**DOI:** 10.1038/s41557-022-01041-9

**Published:** 2022-08-10

**Authors:** Hsin-Yung Yen, Idlir Liko, Wanling Song, Parth Kapoor, Fernando Almeida, Joanna Toporowska, Karolina Gherbi, Jonathan T. S. Hopper, Steven Charlton, Argyris Politis, Mark S. P. Sansom, Ali Jazayeri, Carol V. Robinson

**Affiliations:** 1 Chemical Research Laboratory, University of Oxford; South Parks Road, OX1 3QY, U.K.; 2 OMass Therapeutics Ltd.; the Oxford Science Park, Heatley Rd, Oxford, OX4 4GE, U.K.; 3 Department of Biochemistry, University of Oxford; South Parks Road, OX1 3QU, U.K.; 4 Department of Chemistry, King’s College London, 7 Trinity Street, London, SE1 1DB, UK; 5 School of Life Sciences, Queen’s Medical Centre, University of Nottingham, NG7 2UH, UK; 6 Kavli Institute for Nanoscience Discovery, South Parks Road, Oxford, OX1 3QU, UK

## Abstract

G protein-coupled receptors (GPCRs) signal through cognate G proteins. Despite the widespread importance of these receptors their regulatory mechanisms for G protein selectivity are not fully understood. Here, we present a native mass spectrometry-based approach to interrogate both biased signaling and allosteric modulation of the β_1_ adrenergic receptor (β_1_AR) in response to various ligands. By simultaneously capturing the effects of ligand binding and receptor coupling to different G proteins we probe the relative importance of specific interactions with the receptor through systematic changes in 14 ligands including isoprenaline derivatives, full/partial agonists and antagonists. We observed enhanced dynamics of the ICL3 loop in the presence of isoprenaline, which is capable of acting as a biased agonist. We also show that endogenous zinc ions augment binding in receptor-Gs complexes and propose a zinc ion-binding hotspot at the TM5-TM6 intracellular interface of the receptor-Gs complex. Further interrogation led us to propose a mechanism whereby zinc ions facilitate a structural transition of the intermediate complex towards the stable state.

## Introduction

G protein-coupled receptors are involved in many physiological processes and as a consequence account for nearly 40% of current drug targets. The signaling pathways of these receptors proceed by engaging with four main types of G proteins (G_s_, G_i/o_, G_q_ and G_12_), and in turn, result in different physiological responses. Investigating the mechanisms of receptor G-protein-coupling, and how selectivity is modulated, has become an important quest in the field to understand receptor pathophysiology and to inform better drug design.

Many high-resolution structures of GPCRs have now been solved^1^ primarily from X-ray crystallography, NMR, and more recently with Cryo-EM, to gain insight into the architecture of receptors and the mechanism of their functionality. Mass spectrometry has also been used to understand the ability of different drug molecules or ligands to modulate the structural dynamics of GPCRs, including during complex formation between receptors and G proteins ^2-7^. The utility of mass spectrometry in monitoring ligand binding to GPCRs is compromised by the difficulties encountered in preserving these interactions in the gas phase. However, interactions between a purinergic receptor P_2_Y_1_ receptor and its natural ligand ADP/ATP, or a synthetic ligand, were maintained in a mass spectrometer, while small molecule ligands and peptide binding to the glucagon receptor were enhanced when high concentrations of sodium are present ^8,9^.

The presence of small ligands such as lipids or ions (Na^1+^, Ca^2+^, Zn^2+^) in the structures of GPCRs has been reported and their impact on function and stability of various receptors has been proposed^10-13^. However, it remains challenging to interrogate receptor interactions directly and to illustrate the functional impact of these small molecules due to the absence of a methodology to simultaneously capture the effects of ligand binding and G protein-coupling. In this study, we develop and apply a native mass spectrometry (nMS) approach to interrogate the molecular pharmacology of a purified GPCR, the turkey β_1_-adrenergic receptor (tβ_1_AR). By capturing the G protein-coupling activity of the receptor and monitoring its response to a range of agonists and antagonists, we delineate the propensity of receptor coupling to G_s_ and G_i_ pathways modulated by different ligands. We demonstrate the biased propensity of isoprenaline in stimulating G_i_ protein-coupling and, unexpectedly, endogenous zinc ions which preferentially stabilizes receptor-G_s_ protein complexes. Following in-depth investigation by means of nMS, MD simulation and protein mutagenesis, we reveal a novel allosteric mechanism of zinc ions in coordinating the structural transitions during the formation of receptor-G_s_ protein complexes, enabling us to propose a role for metal ions in modulating the selectivity of G protein-coupling.

## Results

### High levels of G protein-coupling are observed for agonist-bound tβ_1_AR

To develop our approach we selected tβ_1_AR since its molecular pharmacology is well understood. To facilitate protein purification and to retain functionality, we modified a previously described stabilized construct of tβ_1_AR (β44-m23)^14,15^ (see Methods). With the same truncation and deletion on N-termini, C-termini and intracellular loop 3, two reversal mutations (A227Y and L282A) were introduced to enable full activation and high affinity agonist binding of the receptor in the presence of G proteins and nanobodies^16,17^. The mutation R284K, equivalent to the residue of β_2_AR, was included to improve the binding of Nb6B9 nanobody^18^. In addition, glutamic acid at position 130 was mutated to tryptophan (E130W) to allow purification of the receptor in its *apo* state via stabilizing the intrahelical interactions between TM3, TM4 and TM5^19^ cAMP assays confirmed the activity of the engineered receptor in response to various ligands (Extended Data Figure 1a).

**Fig. 1:**
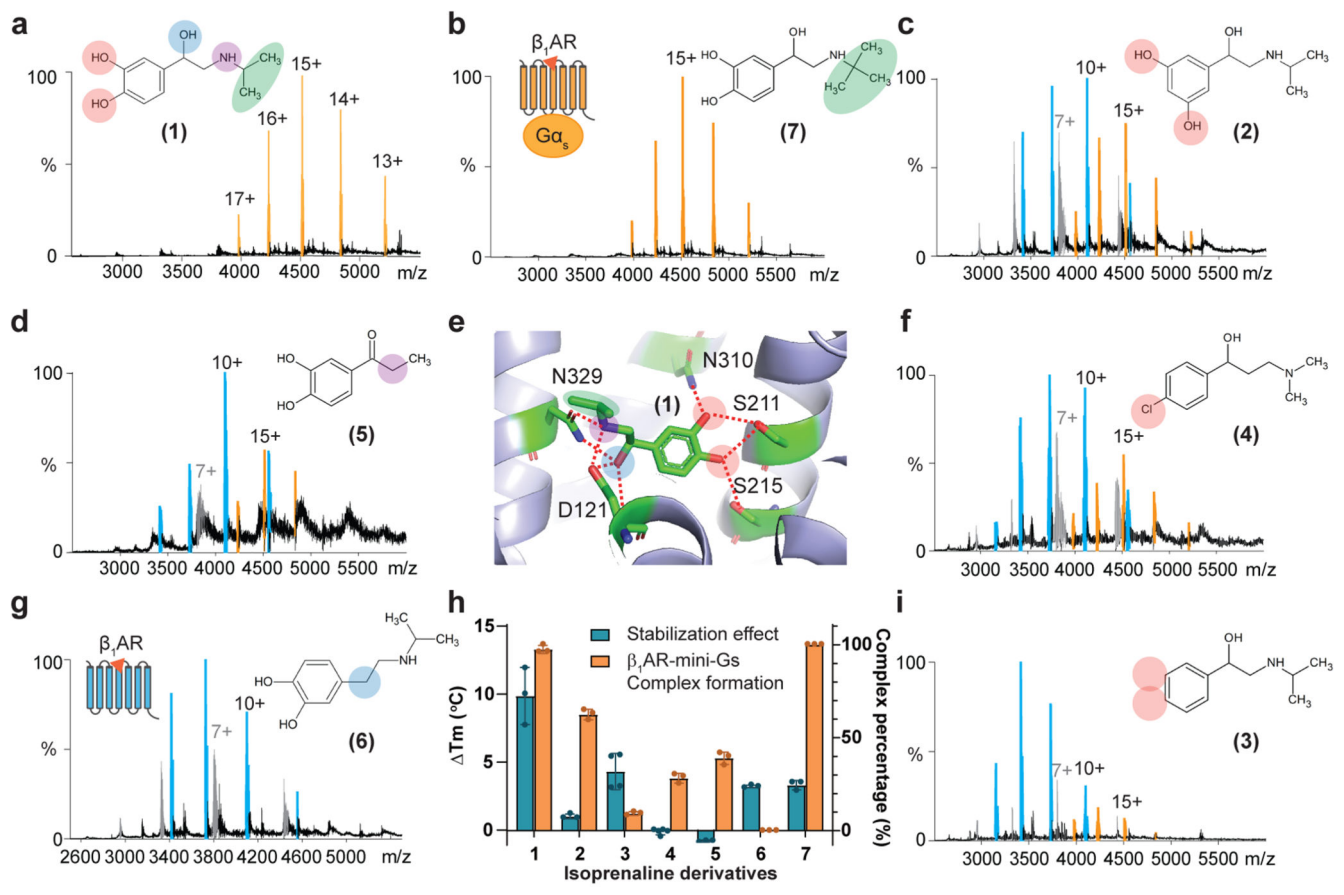
Interrogating the structure-function relationship of isoprenaline derivatives via nMS. **a-g and i,** Representative MS spectra of purified tβ_1_AR (5 μM) in complex with mini-G_s_ (6 μM) in the presence of various isoprenaline derivatives each at a concentration of 250 μM. The peaks assigned to the receptor-mini-G_s_ complex, receptor monomer and mini-G_s_ are highlighted in orange, blue and grey respectively. The structures of isoprenaline derivatives are numbered and illustrated beside each spectrum. **e,** Structure of tβ_1_AR in complex with isoprenaline (PDB 2Y03) depicts the critical interactions between the receptor and isoprenaline, denoted by red dashed lines. **h,** Impact of isoprenaline derivatives on the thermostability (ΔTm °C) and extent of complex formation (tβ_1_AR / tβ_1_AR-mini-G_s_ complex (%)) stimulated by various compounds and assessed by MS. Purified tβ_1_AR was pre-incubated with various isoprenaline derivatives prior to the thermostability assay (see Method) and the degree of stabilization effect of compounds was determined by the difference of protein melting point (ΔTm) in comparison to the untreated condition. The error bars in **h** are plotted as mean ± s.d. from at least three independent experiments

Mass spectrometry measurements of purified tβ_1_AR were optimized (see Methods). In addition to the exact mass of the protein a modification of mass 132.8 ± 0.47 Da is apparent assigned to O-xylosylation (Supplementary Fig. 1). In order to investigate the activity of the purified receptor we next utilized mini-G_s_, the engineered G_α_ subunit which forms a stable complex with active receptors, to examine the extent of receptor coupling in the presence of a saturating concentration of the full agonist isoprenaline **1**. We observed full complex formation (Fig. 1a) whereas previously only ~60% complex formation was detected from isoprenaline co-purified receptor without the E130W mutation^20^. Although the E130W mutation and isoprenaline treatment both significantly increase receptor stability, this result implies a better G protein-coupling activity of this receptor, attributed to stabilization of the TM4-3-5 intrahelical interactions.

### Molecular pharmacology of tβ_1_AR is recapitulated by our nMS platform

To assess the sensitivity of our approach to changes in the structure of the agonist we first investigated six derivatives of isoprenaline that have been well-characterised previously for their ability to attenuate tβ_1_AR G_s_ protein-coupling (Supplementary Fig. 2). X-ray crystal structures of tβ_1_AR bound to isoprenaline highlight the main contacts to the receptor: (catecholamine meta-hydroxyl groups form hydrogen bonds to Ser211, Ser215 and Asn310 and the secondary amine and β-hydroxyl group interact with Asp121 and Asn329) (Fig. 1e). The stimulatory activity of these compounds for tβ_1_AR G_s_ protein-coupling was further examined by our nMS platform. (It is important to note however that native MS of membrane proteins is performed at elevated energies to remove bound detergents, which often leads to removal of weakly bound ligands, which are then not observed in mass spectra. Importantly, however, we capture the effect that ligand binding has in solution, and consequently on complex formation). We first tested orciprenaline **2** and 1-phenyl-2[(propan-2-yl)amino]ethan-1-ol **3**, which abolish the interactions between the catecholamine meta-hydroxyl and the receptor. The change of the meta-hydroxyl group from position 3 to 4 in **2** results in ~60% reduction of mini-G_s_-coupling (Fig. 1c). Removal of both meta-hydroxyl groups in **3**, led to 90% attenuation of coupling aligning with previous observations that the two conserved serine residues on TM5 are crucial for agonism (Fig. 1i)^14^. Introduction of a chloride, at position 3 in **4**, partially recovered the coupling activity. Although a chloride is expected not to retrieve the hydrogen bonding, its strong electronegativity may contribute the formation of the π interactions between the phenyl ring of isoprenaline and adjacent serine residues of receptor (Fig. 1f). Examining the impact of the secondary amine and β-hydroxyl group with two derivatives **5** and isopropyldopamin **6** we found 75% attenuation and no coupling respectively (Fig. 1d and 1g). Intriguingly, our MS platform is able to detect the stimulatory activity of **4** and **5** which are expected to have low binding-affinity to the receptor, highlighting the sensitivity of MS platform.

While mass spectrometry is able to detect the downstream effects of these isoprenaline derivatives it is challenging to observe direct binding under the detergent micelle conditions used here, as outlined above. To confirm the binding of these derivatives to the receptor we therefore performed an orthogonal assay in which we assessed the thermostability increase achieved from ligand binding to the receptor. We found that incubation of isoprenaline significantly improved receptor stability whereas most derivatives stabilized the receptor to a lesser extent (Fig. 1h), showing a certain degree of interactions between derivatives and receptor can be preserved. However, we did not observe any stabilization effect of two derivatives, 1-(4-chlorophenyl)-3-(dimethylamino)propan-1-one hydrochloride **4** and 3,4 dihydroxypropiophenone **5** although weak agonist activity was observed using native MS. Comparison across the ligand structures allows us to deduce that the modifications in **6** retain binding competency but completely lose their stimulatory effect, whereas **5** remains active but compromises most of its binding to the receptor. Moreover, the tri-methyl group on colterol **7** retained the same full-coupling activity as **1** (Fig. 1b).

**Fig. 2:**
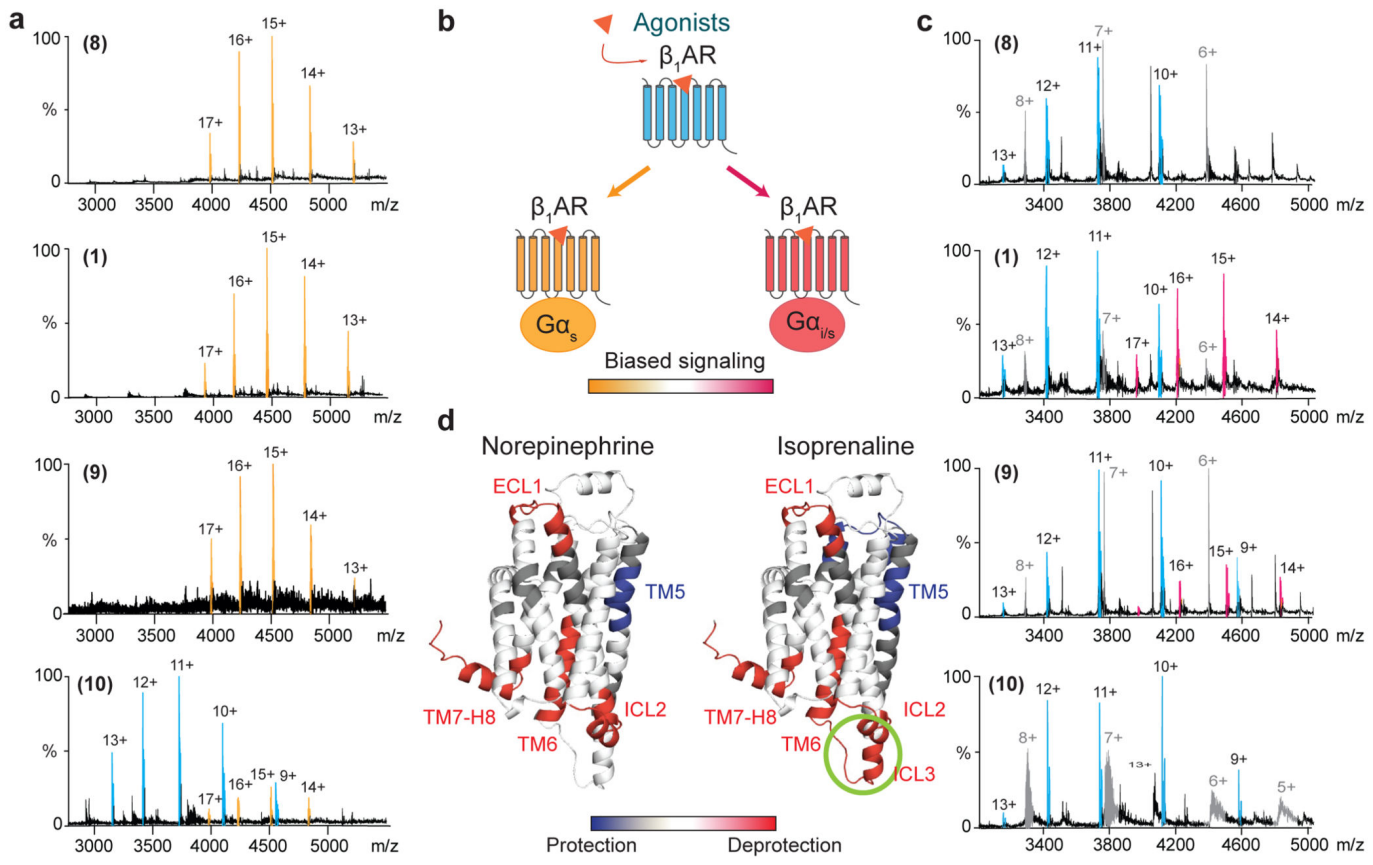
Characterizing the coupling of tβ_1_AR coupling to engineered Gα subunits. **a,** MS spectrum of purified tβ_1_AR in complex with mini-G_s_ in the presence of various agonists (Isoprenaline **1**; Norepinephrine **8**; Carmoterol **9**; Dobutamine **10**) at a concentration of 25 μM. The peaks assigned to receptor-mini-G_s_ complex and the receptor monomer are denoted in orange and blue respectively. **b,** Schematic illustration of the stimulation propensity of agonists toward G_s_ and G_i_ proteins and the potential biased effect of agonists. **c,** Mass spectra of tβ_1_AR-mini-G_i/s_ complexes formed in response to various agonists at 25 μM. The signal of tβ_1_AR-mini-G_i/s_ complex, receptor monomer and mini-G_s_ are in magenta, blue and grey respectively. **d,** Different deuterium uptake upon tβ_1_AR activation in the absence of G-proteins is induced by norepinephrine and isoprenaline and mapped on the structure of tβ_1_AR (2Y03). The increased uptake in comparison to receptor without compound treatment is denoted in red, whereas decreased uptake is colored blue. The ICL3 motif is uniquely modulated by isoprenaline is highlighted (green circle). Representative spectra from three independent experiments are shown in **a** and **c**.

To further establish our native-MS platform for molecular pharmacology characterization of full agonists (norepinephrine **8,** carmoterol **9** and **1**); partial agonists (dobutamine **10** and salbutamol **11)** and antagonists (cyanopindolol **12**, carazolol **13** and carvedilol **14**) we added these well-characterised ligands individually to solutions containing the receptor and mini-G_s_. Complete complex formation was observed for the full agonists **8, 9** and **1**, at a saturation concentration, while the two partial agonists **10** and **11** elicited only a limited response under the same MS conditions (Fig. 2a and Supplementary Fig. 3). Little or no complex formation was detected for antagonists **12** and **13** respectively; **12** is reported to be a weak agonist^21^. The sensitivity of MS detection, to low populations of complexed receptor, highlights our ability to provide a direct readout (Supplementary Fig. 3). Quantifying the extent of complex formation allowed us to plot dose-response curves for agonists and partial agonists which are comparable to EC50 values reported previously, with the same rank order ^22^. Addition of antagonist **13** led to the expected dose-dependent right shift in the isoprenaline curve confirming the antagonistic action of **13** (Supplementary Fig. 3).

### Capturing biased signaling via G_i_ protein-coupling

To explore the stimulatory propensity of drug molecules toward different G proteins, we carried out analogous experiments to those above for mini-G_i/s_ (the H5 motif of mini-G_s_ was replaced with the sequence of G_i_ (Fig. 2b and Fig. S4a)). β-adrenergic receptors have previously been reported to bind selectively to G_i_ proteins under certain stimulatory conditions^23,24^, suggesting a “switch” mechanism to propagate different signaling events. Intriguingly, for the three full agonists **1, 8** and **9**, isoprenaline **1** induced mini-G_i/s_-coupling to a greater extent than **9**, whereas no complex formation was detected with the natural ligand **8** (Fig. 2c). The propensity of isoprenaline to stimulate G_i_ protein-coupling of tβ_1_AR was further validated by a competition experiment in which the receptor was incubated with mini-G_s_ and mini-G_i/s_ at equimolar ratios. In line with the preferential G_s_ signaling of tβ_1_AR, significantly more coupling between the receptor and mini-G_s_ was observed compared with mini-G_i/s_ in a competitive manner. **1** was able to induce receptor complex formation with both mini-G_s_ or mini-G_i/s_ whereas no receptor-mini-G_i/s_ complex was detected in the presence of the other full agonists **8** and **9** (Supplementary Fig. 4). These data are supported by the higher efficacy of isoprenaline compared with norepinephrine in triggering the dissociation of trimeric G_i_ proteins^25^, and indicates the propensity of isoprenaline to stimulate G_i_ protein-coupling of tβ_1_AR.

To understand the structural changes associated with the isoprenaline-mediated biased effect, we performed hydrogen deuterium exchange mass spectrometry (HDX-MS) on the receptor alone to investigate its conformational dynamics in the presence of the natural ligand norepinephrine **8** or isoprenaline **1**. The overall differences in deuterium exchange of the receptor upon activation induced by these two agonists was very similar with increased deuterium uptake on the motifs of ECL1, ICL2, the intracellular tip of TM6, and TM7-H8^17,26^ (Fig. 2d and Supplementary Fig. 5). TM5 however exhibits more protection in both cases following activation, consistent with a reduction in mobility upon ligand binding to the motif 210-218 of TM5. Moreover isoprenaline **1**, but not norepinephrine **8**, specifically promoted higher deuterium uptake of ICL3, consistent with increased conformational dynamics. We propose that the increased dynamics of this loop likely plays a role in promoting biased signaling pathways wherein alternative conformations might be facilitated.

### Endogenous zinc ions stabilize G_s_ protein-coupling

**Fig. 3:**
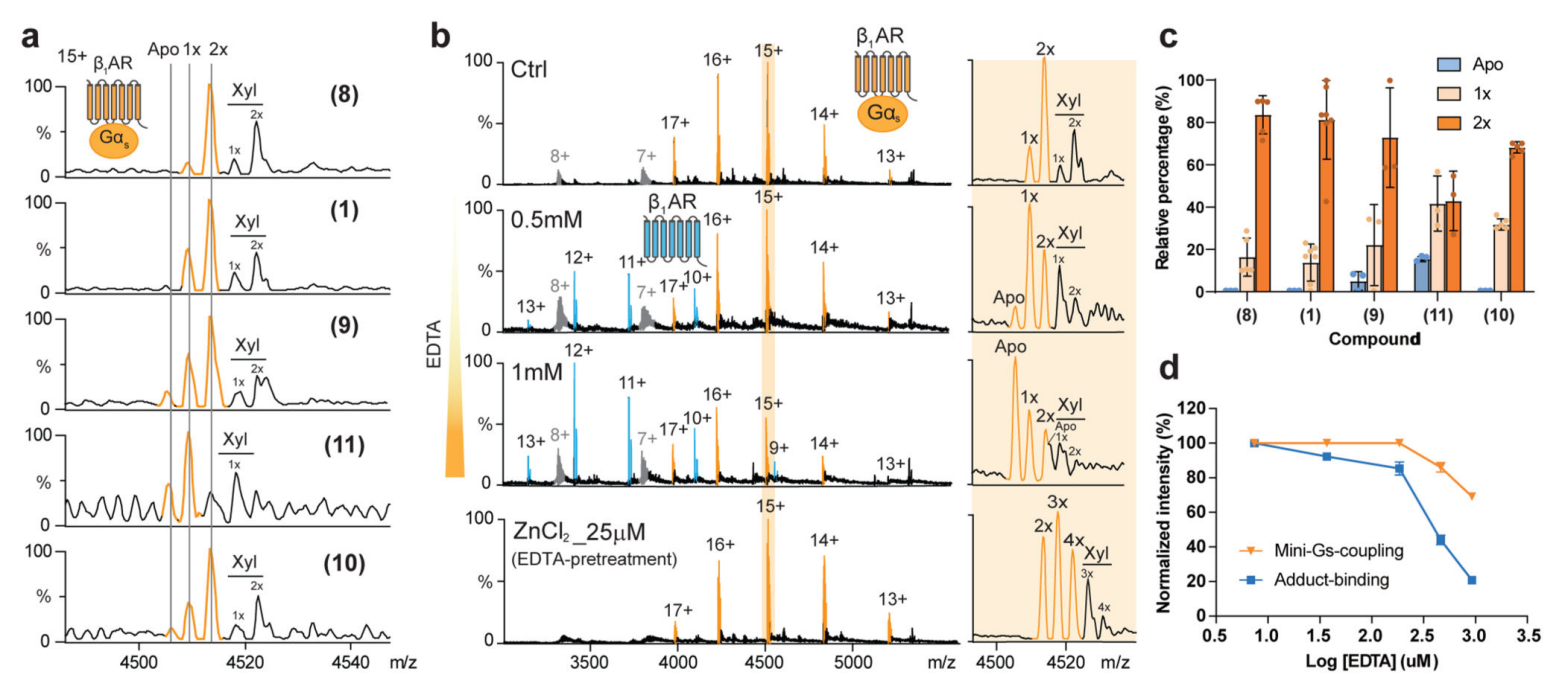
Identification of an endogenous metal in tβ_1_AR-mini-G_s_ complex and its functional relation to G_s_ protein-coupling. **a,** Endogenous metal adducts were detected in tβ_1_AR-mini-G_s_ complex under stimulation with different agonists (Isoprenaline **1**; Norepinephrine **8**; Carmoterol **9**; Dobutamine **10**; Salbutamol **11**). The stoichiometry of metal-binding is denoted on the individual peak (1x: one adduct, 2x: two adducts). **b,** The impact of EDTA on tβ_1_AR-mini-G_s_ complex formation and its association with the endogenous metal. The binding stoichiometry of the metal ligand was denoted (1x-4x, one to four adducts). Supplement of exogenous ZnCl_2_ at 25 μM into EDTA pretreated receptor and mini-G_s_ recovered the activity of complex formation of tβ_1_AR and mini-G_s_ (bottom spectrum). The peaks assigned to the receptor-mini-G_s_ complex, receptor monomer and mini-G_s_ are highlighted in orange, blue and grey respectively**. c,** Percentage of endogenous metal adduct in different stoichiometries normalized to the total intensity of tβ_1_AR-mini-G_s_ complex in response to different tβ_1_AR agonists. The bars are plotted as mean ± s.d. from the number of independent experiments (n=5 for Norepinephrine **8** and Dobutamine **10**; n=7 for Isoprenaline **1**; n=3 for Carmoterol **9** and Salbutamol **11**). **d,** Quantification of tβ_1_AR-mini-G_s_ complex formation and its association with the metal ligand in the presence of EDTA at different concentrations. The relative percentage was calculated by normalizing the condition without EDTA. The curves are plotted as mean ± s.d. from three independent experiments.

While investigating the complex formation between tβ_1_AR and mini-G proteins, we observed an adduct with a mass of 64.2 ± 1.4 Da present in all receptor-mini-G_s_ complexes. The binding stoichiometry of this adduct (1 or 2), which we tentatively assign as zinc or copper ions based on mass, was found to vary with different agonists. For the natural ligand norepinephrine **8** the predominant population of the complex contains two metal ions. The absence of a statistical distribution of ligand-bound species implies specific ion binding and implies a potential role for this endogenous ligand in modulating G protein-coupling (Fig. 3a and 3c). Given the measured mass of this ligand, tentatively identified as zinc or copper ions, we treated the receptor complex solution with EDTA to chelate divalent cations and monitored the impact of this chelation on G protein-coupling (Fig. 3b). A significant reduction in the levels of receptor in complex with the adduct was observed together with lower levels of the receptor/mini-G_s_ protein complex (Fig. 3b and 3d). We anticipated that this adduct maybe Zn^2+^ ions given the observation of their effect in a previous cell-based study ^27^ although molecular details were not ascertained. To confirm the presence of Zn^2+^ ions, we conducted Inductively Coupled Plasma Mass Spectrometry (ICP-MS) to examine the trace elements in our protein solutions. Intriguingly, very low amount of zinc, < 5 ppb, was detected in the purified receptor solution but no zinc ions were detected in the buffers. The divalent zinc ions must therefore be endogenously sourced from the cell lines used for receptor overexpression (Supplementary Fig. 6). Given the diminutive amount of zinc ions, this result highlights the sensitivity of nMS for detecting direct interactions between the receptor and Zn^2+^ ions. Moreover, addition of exogenous zinc ions to EDTA-pretreated receptor and mini-G_s_ led to full recovery of receptor/mini-G_s_ complex formation (Fig. 3b). High levels of Zn^2+^ led to the formation of multiple zinc ion adducts that are likely to be the result of non-specific binding. Importantly, addition of copper ions did not alter receptor/mini-G_s_ complex formation significantly (Supplementary Fig. 7). Moreover, reduction of the concentration of agonists, isoprenaline and carmoterol (5-fold less than receptor (Supplementary Fig. 3)) the population of receptor-mini-Gs complex was significantly reduced but the complex retained preferential binding to zinc ions. Together these experiments align with our hypothesis that zinc ions serve as an essential allosteric modulator for G protein-coupling even at lower (not saturating) ligand concentrations.

**Fig. 4:**
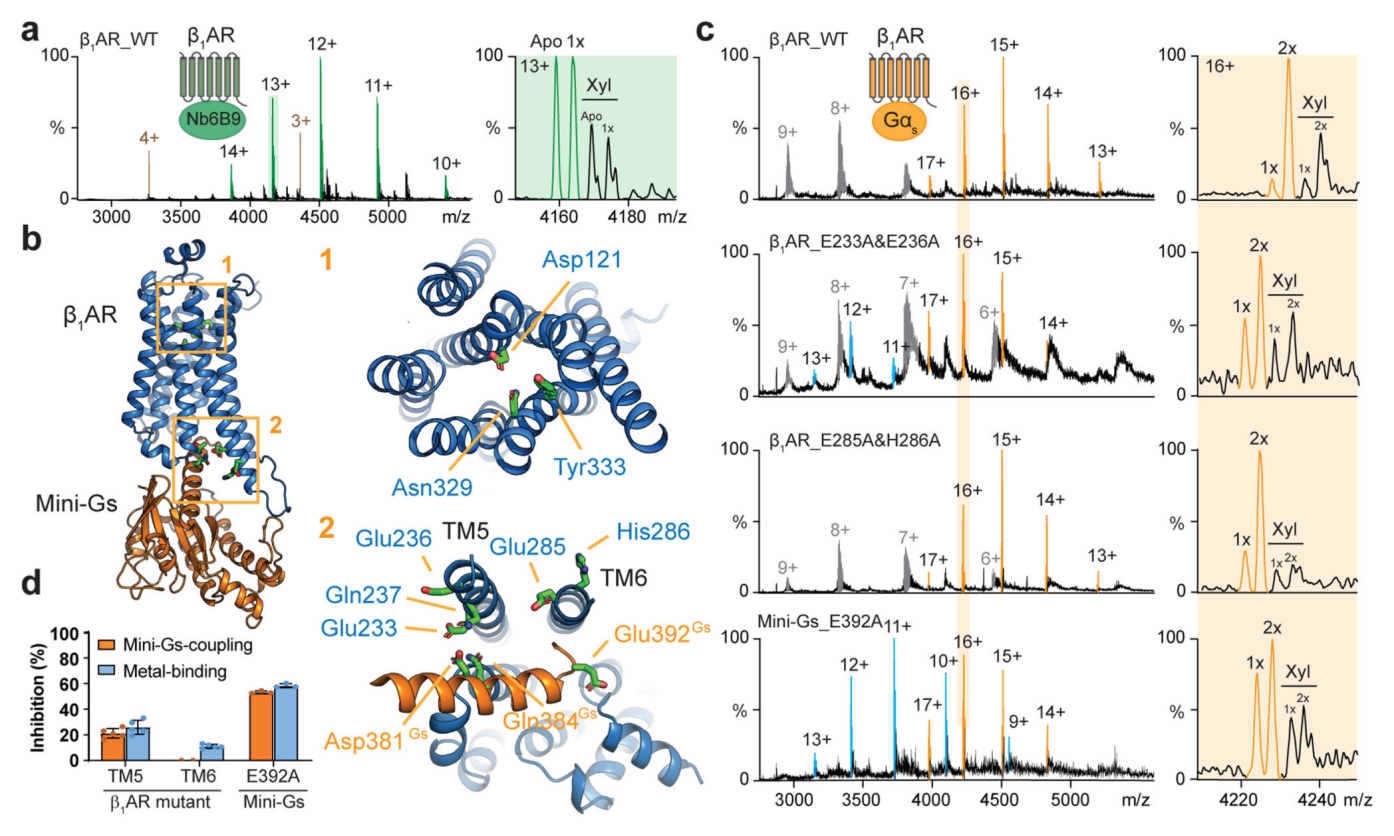
Localization of zinc-binding hotspots and their functional impact on G_s_ protein-coupling. **a,** MS spectrum of tβ_1_AR-Nb6B9 complex indicates reduced binding of endogenous metal. The complex is highlighted in green and its metal-binding stoichiometry reduced to first order (denoted as 1x). The peaks assigned to Nb6B9 alone are highlighted in brown. **b,** Molecular dynamics simulation of the tβ_1_AR-mini-G_s_ complex, in the presence of zinc, revealed two zinc-binding hotspots. The zinc contacts in the orthosteric ligand-binding site (Asp121, Asn329 and Tyr333) are shown in the upper box, whereas a subset of the zinc contacts in the intracellular interface are highlighted on the receptor (Glu233, Glu236, Gln237, Glu285 and His286) and mini-G_s_ (Asp381, Gln384 and Glu392) in the bottom box. **c,** The impact of mutations to the zinc contacts on tβ_1_AR-mini-G_s_ complex formation and metal-binding (orange boxes). Two receptor variants (E233A&E236A and E285A&H286A) and a mini-G_s_ mutant (E392A) were examined. Representative spectra from three independent experiments are shown. Receptor monomer and the receptor-mini-G_s_ complex are shown in blue and orange respectively. **d,** The inhibitory effect of zinc contact mutants on tβ_1_AR-mini-G_s_ complex formation (orange bar) and metal-binding (blue bar). The percentage of inhibition was calculated for the results acquired from wild-type tβ_1_AR and mini-G_s_ as shown in the top spectrum of **c**. Bars show mean ± s.d. from three independent experiments by the same batch of purified proteins.

In order to gain structural insights into possible zinc interactions with tβ_1_AR, we carried out molecular dynamic simulations at atomic resolution for tβ_1_AR, in either inactive or active conformations, in a lipid bilayer with Zn^2+^ ions present in the aqueous phase (Supplementary Fig. 8). The simulation revealed dynamic zinc interactions with the receptor at two putative areas: on the extracellular motifs one surrounding the orthosteric ligand-binding site and the other corresponding to the cytoplasmic interface of the TM3, TM5 and TM6 helices. Given that zinc interacts with the receptor cytoplasmic interface, where the conformational movement plays a critical role for G protein-coupling, we next examined Zn^2+^ -binding with tβ_1_AR-mini-G_s_ complex (Fig. 4b). Overall the Zn^2+^-binding contacts are similar to the results for the receptor alone; residues Asp121, Asn329 and Tyr333 from the orthosteric pocket and Glu233, Glu236, Gln237, Glu285 and His286, from the cytoplasmic interface, form interactions with zinc. Intriguingly, we observed residues of mini-G_s_, Asp381, Gln384, Glu392 on the H5 helix and Asp354 on α4-β6 loop, additionally contribute to binding which is suggested to form more stable contacts with Zn^2+^ (Fig. S8), implying the potential role of Zn^2+^ in stabilizing G protein-coupling.

To probe the possible involvement of the Zn^2+^ binding sites observed in β_1_AR function, we utilized a nanobody (Nb6B9), which specifically recognizes the active receptor. Given that structures of receptors bound to Nb6B9, or to mini-G_s_, are virtually identical (root mean square displacement (r.m.s.d.) = 0.4–0.6 Å) ^18,28^, the utility of Nb6B9 provides an appropriate reference to investigate the impact of G protein-coupling on zinc-binding. Compared to the receptor-G protein complex, the spectrum of the tβ_1_AR-Nb6B9 complex, following stimulation with isoprenaline, revealed significantly less Zn^2+^ binding (Fig. 4a). This observation indicates the presence of a G protein-specific zinc-binding site in the intracellular interface between the receptor and G protein.

To further interrogate the contact residues which are important for the effect of Zn^2+^ on G protein-coupling, we generated two tβ_1_AR variants with alanine mutations on TM5 (E233A and E236A) and TM6 (E285A and H286A) motifs, respectively. The results reveal that the TM5 mutant attenuates endogenous zinc-binding and coupling activity by 25.8 ± 5.4% and 21.2 ± 3.8% respectively, whereas the TM6 mutant shows only a slight reduction (11 ± 1.56%) on endogenous zinc-binding (Fig. 4c and 4d). Our results are distinct from previously published data that mutation of Glu225 of β_2_AR (equivalent residue to Glu233) did not abolish the allosteric effect of zinc on the cAMP response observed from cell-based assays^27^. To exclude the potential adverse effect of mutations on receptor conformation, we examined the binding activity of Nb6B9 to each mutant. The mutations had no effect on agonist induced receptor/nanobody complex formation (Supplementary Fig. 9). Our results imply therefore that the attenuated activity observed in the TM5 mutant is G_s_ protein-specific and likely caused by reduced Zn^2+^ binding.

We next explored the binding contact on mini-G_s_ by introducing an alanine mutation on mini-G_s_ (Glu392). This point mutation resulted in a significant decrease in both endogenous Zn^2+^ and receptor/G protein interaction by 58.3 ± 1.57% and 53.1 ± 1.1% respectively (Fig. 3c and 3d). We further utilized a potential mean force (PMF) calculation to examine the effect of zinc-binding on the free-energy landscape of interactions between tβ_1_AR and mini-G_s_. The result indicates that zinc-binding at the interface of the complex stabilizes the association by ~15 kJ mol^−1^ relative to the *apo* state (Supplementary Fig. 9). Collectively, our results suggest the endogenous zinc ion functions as a positive allosteric modulator (PAM) of G_s_ protein-coupling through stabilization of the interface between the receptor and G_s_ protein.

### Endogenous zinc facilitates the structural transition of G_s_ protein-coupling

**Fig. 5:**
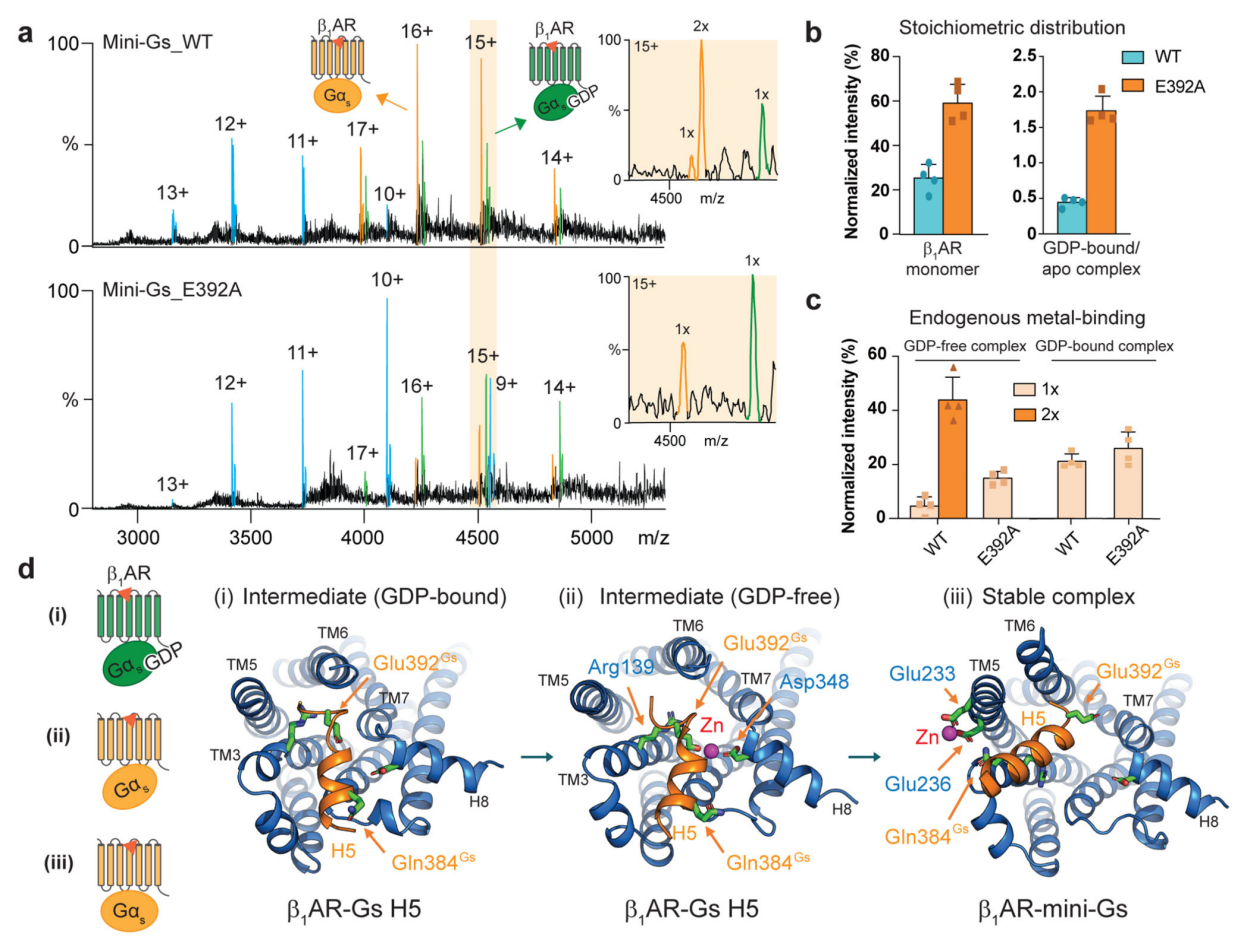
The allosteric effect of endogenous zinc on the selectivity of mini-G_s_. **a,** Representative MS spectra of tβ_1_AR coupling to mini-G_s_ wild-type (WT) and E392A mutant in response to isoprenaline at 25 μM. The GDP-bound and GDP-free complex are highlighted in green and orange, respectively, whereas receptor monomer is in blue. The binding stoichiometry of metal ligand is denoted in the orange boxes. **b,** Relative percentage of stoichiometric distribution of tβ_1_AR monomer and tβ_1_AR-mini-G_s_ GDP-bound and GDP-free complexes. The results indicate weaker complex formation and slower structural transition to the stable state in mini-G_s_ E392A. **c,** Binding stoichiometry of the metal ligand in the intermediate and stable complex of tβ_1_AR-mini-G_s__WT or tβ_1_AR-mini-G_s__E392A. Bars in **b** and **c** show mean ± s.d. from three independent experiments by the same batch of purified proteins. **d,** Schematic representation of the hypothesized activation mechanism of tβ_1_AR-G_s_ complex formation according to molecular dynamic simulations of tβ_1_AR-G_s_ H5 peptide and tβ_1_AR-mini-G_s_ complexes. Receptor first forms contacts with G_s_ protein in a GDP-bound state (i) as an intermediate complex it further transits to a conformation where zinc interacts both Glu392^Gs^ and Asp348 ^β1AR^ (ii) leading to the formation of stable complex in a GDP-free state (iii). Snapshots of the MD structures illustrate the spatial orientation of the H5 motif of G_s_ protein (orange) and its coordination with Zn^2+^ in the discrete conformations of the complex.

The potential involvement of Gα_s_-specific residues Gln384 and Glu392 in Zn^2+^ binding, and our observation of reduced zinc-binding in the tβ_1_AR-mini-G_i/s_ complex (Fig. S4d), suggests that endogenous zinc might modulate the selectivity of G proteins. A structure reported for β_2_AR in complex with the peptide of Gα_s_ H5 motif addressed the role of Gα_s_ Glu392 in the formation of receptor-G_s_ complex in the GDP-bound state^29^. That study presented an intermediate configuration of complex formation where Arg389 and Glu392 on Gα_s_ contact Thr68, Arg131 and Tyr141 of β_2_AR. To investigate the potential for Zn^2+^ to influence this intermediate complex stage, and thereby the selectivity of G_s_ protein coupling, we performed MS experiments with mini-G_s_ coupling to tβ_1_AR with very short incubation times (t < 1 min). The tβ_1_AR-mini-G_s_ complex was detected immediately after adding isoprenaline, indicating the fast kinetics of complex formation. Intriguingly, in addition to the GDP-free complex, we observed the presence of the GDP-bound complex, aligning with the hypothesis of intermediate complex formation (Fig. 5a). We next examined the coupling of mini-G_s_ with the E392A variant under the same experimental conditions, again with very short incubation times (t < 1 min), and observed 35% reduction in complex formation in comparison to the wild-type. Furthermore, the intensity ratio between GDP-bound and GDP-free complex was increased significantly for the E392A mutant, indicating a slower transition rate from the intermediate to the stable state (Fig. 5a and 5b).

Of interest the extent of endogenous zinc-binding in mass spectra reduced dramatically in the mini-G_s_ E392A mutant-tβ_1_AR complex following short incubation times compared to longer incubation (83.1% vs 53.1%). By contrast no significant difference was observed between the mutant and wild-type for metal ion binding in the GDP-bound state (Fig. 5a and 5c). We infer therefore that G_s_-Glu392 is not involved in zinc binding at the GDP-bound complex stage, prior to structural rearrangement of the nucleotide-binding pocket of G_s_.

To further investigate the role of Glu392 in complex formation we carried out atomic simulations for the tβ_1_AR-G_s_ H5 complex in the intermediate configuration and revealed a further conformational state where zinc ions bind to Glu392 of G_s_ H5 and Asp348 of the receptor. The position of this zinc suggests that it induces an upward rotation of G_s_ H5 which could be important structurally for the transition of the tβ_1_AR-G_s_ intermediate complex to the stable state (Fig. 5d), as seen in the analogous β_2_AR-trimeric G_s_ complex. In addition to the Glu392 mutation on mini-G_s_, we examined the coupling activity of tβ_1_AR D348A mutant and its association with endogenous metal (Supplementary Fig. 10). The mutation on Asp348 at position 8.49 (Ballesteros-Weinstein numbering scheme) unexpectedly increases the extent of complex formation with mini-G_s_. This observation coincides with previous studies in that the ionic interactions between negatively charged glutamic acid (E^8.49^) and positive residue arginine (R^2.46^) are important for stabilizing the receptor in an inactive conformation^30,31^. The structure of inactive tβ_1_AR (PDB_2VT4) also presents a similar ionic lock between Asp348 and Arg71; hence mutation of Asp348 is suggested to destabilize the inactive receptor, in line with our results. Although it is not possible to use this variant to validate the role of Asp384 in structural transition of the intermediate complex, the binding of endogenous metal to tβ_1_AR D348A is substantially increased in its monomeric state, highlighting the functional correlation of metal-binding to receptor activity (Supplementary Fig. 10).

## Discussion

Herein, we have demonstrated a novel MS approach for investigating the pharmacology of GPCRs. Combining the sensitivity and near atomic mass resolution of the mass spectrometer, whilst preserving the receptor-G protein interactions, renders our methodology capable of monitoring, with very high sensitivity, the full spectrum of receptor pharmacology *in vitro*. In turn this has led to the discovery of endogenous zinc as an allosteric modulator of receptor activation. Overall our results allow us to propose that fine tuning of agonists, antagonists and allosteric modulators can be captured on one platform with results interpreted with the aid of MD simulation, HDX-MS, site directed mutagenesis and the plethora of high-resolution structures of GPCRs. That endogenous zinc facilitates the structural transition of the receptor-G_s_ complex from the intermediate (GDP-bound) to the stable (GDP-free) state. Given that the interaction of G_s_ Glu392 with zinc triggers the upward rotation of the H5 motif and that Glu392 is largely solvent-exposed in the stable complex, with no specific interaction observed with the receptor (Supplementary Fig. 10), its interaction with endogenous Zn^2+^ may play a critical role in modulating the selectivity of G_s_.

Understanding of GPCR agonism and allostery has been emerging in recent years^32^. The conformational plasticity of receptors reflects the versatility of ligands in modulating receptor activity. Multiple allosteric sites, ranging from the extracellular and transmembrane domains to the cytoplasmic face, have been described for various receptors^33^. However, it is challenging to predict receptor allostery due to an absence of conserved allosteric sites and mechanistic insight of their action. The MS approach presented here to interrogate the receptor pharmacology in high definition and the discovery of endogenous Zn^2+^ ions as an allosteric modulator exemplifies the utility of this platform. Moreover, the involvement of H5 of the Gα subunit in the allosteric regulation highlights the role of the G protein itself in receptor allostery. A similar phenomenon was observed previously for a specific lipid PtdInsP_2_ which improves the activity of coupling via bridging between the receptor and the G protein^20^. The data presented here offer an enhanced understanding of receptor/G protein interaction regulation. Given the zinc coordinates with the selectivity determinant of G_s_, the potential to regulate its binding or to mimic its mechanism by high potency small molecules may provide a new avenue for modulating the kinetics of specific receptor/G protein signaling for therapeutics purposes. Overall, our study offers a novel perspective for rational design of GPCR agonists and allosteric modulators.

## Methods

### Constructs and proteins

Expression plasmid for a thermostabilized variant of turkey (M. gallopavo) β_1_AR was used in our experiments. The synthesized cDNA encoding tβ_1_AR, flanked with N-terminal Flag tag and Strep tag, and C-terminal His tag, was cloned into pFastBac™ 1 vector via NheI and NotI cloning sites. The protein sequence ranges from 44-364 with intracellular loop 3 (ICL3) truncation (244-272), and contains 7 thermostabilizing point mutations (R68S, C116L, E130W, D322K, F327A, F338M, C358A). Purified β_1_AR, engineered Ga_s_ (mini-G_s_) and Nanobody Nb6B9 were utilized for mass spectrometry analysis.

### Expression and purification of mini-G_s_ and mini-G_i/s_

The engineered minimal G protein, mini-G_s_ construct R414 and mini-G_i/s_ construct R43 cloned into pET24a vector were expressed in *E*. *coli* BL21(DE3) strain and purified by Ni^2+^ affinity chromatography, followed by cleavage of the histidine tag using TEV protease. The cleaved tag, protease and undigested mini-G proteins were removed by reverse IMAC purification on Ni^2+^-NTA. Proteins were concentrated to 2 mg/ml in 20 mM HEPES, pH 7.5, 100 mM NaCl, 10% v/v glycerol, 1 mM MgCl_2_, and 10 mM GDP.

### Expression and purification of tβ_1_AR

The construct of tβ_1_AR was over-expressed in insect cells using Bac-to-Bac^®^ Baculovirus expression system (Thermo Fisher). The recombinant baculoviruses prepared using the expression vector pFastBac 1 (Thermo Fisher) were applied to infect Sf9 cells (Invitrogen, 11496015) with the Multiplicity of Infection (MOI) between 1-2.The cell membrane was enriched and solubilized in 20 mM Tris-HCl pH8, 350 mM NaCl, 3 mM imidazole, 1.5% (w/v) n-dodecyl-β-D-maltopyranoside (DDM, Anatrace) for 15 mins. The supernatant was isolated by ultra-centrifugation at 175,000 x g for 1 hr and applied to HiTrap TALON crude column (GE healthcare) for affinity enrichment. The column was washed by ten column volumes of 20 mM Tris–HCl pH 8, 350 mM NaCl, 3 mM imidazole and 0.05% DDM after loading the supernatant, and receptor was eluted by a gradient of 20 mM Tris–HCl pH 8, 350 mM NaCl, 250 mM imidazole and 0.05% DDM in three column volumes. The pH of all buffers were adjusted at room temperature. The fractions containing receptor were pulled and concentrated to the final concentration 2-3 mg/ml via Amicon^®^ centrifugal filter of molecular weight cut-off 50 kDa for following applications. In order to mitigate the experimental variations which may cause the differential binding with endogenous zinc ions, we carefully controlled our experimental conditions during purification of the various tβ_1_AR mutants. Specifically, the quantity of starting biomass (20 g), ratio between cell membranes and detergents (DDM: membrane proteins = 3:1 (w/w)), duration of detergent solubilization (15 mins) and FPLC conditions were strictly controlled.

### Expression and purification of nanobody Nb6B9

The expression gene of Nb6B9 was cloned into the plasmid pET-26b(+)^34^ which contains a N-terminal His-tag followed by a thrombin protease cleavage site. Protein was overexpressed in E. coli strain BL21(DE3) (Agilent Technologies) and purified from the periplasmic fraction was by Ni^2+^ affinity chromatography. The His-tag was removed with the use of a thrombin protease (Sigma) before concentration to 20 mg/ml.

### Non-denatured mass spectrometry of tβ_1_AR

Purified β _1_AR was buffer exchanged into 200 mM ammonium acetate buffer pH 7.4 containing the mixed micelle preparation (DDM: Foscholine16: CHS = 20: 2: 3 (w/w/w)) optimized for GPCR analysis as described previously^8^ before MS analysis by a modified Q-Exactive mass spectrometer (Thermo)^35^. The capillary voltage (1.1 kV) was applied during nano-electrospray, and an optimized acceleration voltage (120 V) was then applied to the HCD cell to remove the detergent micelle from the protein ions, following a gentle voltage gradient (injection flatapole, inter-flatapole lens, bent flatapole, transfer multipole: 7.9, 6.94, 5.9, 4 V respectively). For analyzing receptor complex formation with mini-G_s_, the optimized voltage was applied to the in-source fragmentation (100 V) and HCD cell (100 V) with the same voltage gradient for ion transmission. Spectra were acquired and averaged with a noise level parameter of 3. Backing pressure was maintained at ~0.9 x 10^-9^ mbar. Data was analyzed using Xcalibur 2.2 and the relative percentage of tβ_1_AR in different binding stoichiometry was quantified by UniDec software^36^. The measurement error was derived from the deviation of peak centroids of different charge states corresponding to the same mass species.

### Mini-G_α_ and Nb6B9 coupling to tβ_1_AR

Effector coupling to tβ_1_AR was analyzed by a modified Q-Exactive mass spectrometer after incubating purified tβ_1_AR with mini-G_α_/Nb6B9 at 1:1.2 molar ratio at 4 °C in the coupling buffer (10 mM HEPES, 10 mM Tris-HCl, pH7.4, 200 mM NaCl, 1mM MgCl_2_, 5 mM GDP and 0.05% DDM) containing 25 μM agonists for at least 20 mins. To strip the exogenous metal ligand, both purified tβ_1_AR and mini-G_s_ were pre-treated with 5mM EDTA for 5 mins at 4 °C and then buffer-exchanged into EDTA-free buffers for tβ_1_AR (20 mM Tris–HCl pH 8, 350 mM NaCl and 0.05% DDM) and mini-G_s_ (20 mM HEPES, pH 7.5, 100 mM NaCl, 1 mM MgCl_2_, and 10 mM GDP), respectively. The relative percentage of effector coupling was quantified by UniDec software and the degree of effector coupling was calculated by normalizing the relative percentage of complex to the sum of the percentage of receptor monomer and complex. To examine the inhibitory effect of antagonist, purified tβ_1_AR was pre-incubated with carazolol at desired concentration for 10 mins at 4 °C, followed by the same procedure described above for mini-G_s_ coupling in the presence of isoprenaline. All data analysis was carried out using GraphPad Prism 7 (GraphPad).

To investigate the intermediate complex formation, purified tβ_1_AR and mini-G_s_ were buffer-exchanged into 200 mM ammonium acetate buffer pH 7.4 containing the mixed micelle preparation and 5 mM GDP. tβ_1_AR was pre-mixed with mini-G_α_ at 1:1 molar ratio at 4°C and the protein mixture was introduced into mass spectrometry immediately after adding isoprenaline to final concentration 25 mM. Spectra were acquired for 1 min and the relative percentage of tβ_1_AR monomer, tβ_1_AR-mini-G_s_ intermediate and stable complexes was quantified by UniDec software.

### Nano differential scanning fluorimetry (nanoDSF) for stability measurement of purified tβ_1_AR

tβ_1_AR was diluted to 0.4 mg/ml in protein buffer (20 mM Tris–HCl pH 8, 0.35M NaCl, 3 mM imidazole and 0.05% DDM). β_1_AR compounds such as agonists, agonist derivatives, antagonists and partial agonists were tested at concentrations 100 mM to measure their impact on the receptor stability. The DMSO concentration was maintained at 5% in the final reaction volume, and the control experiments including protein alone, protein in 5% DMSO and compounds alone were conducted as the appropriate references for measuring stabilization effect of compounds. Protein sample and compounds were mixed at a fixed molar ratio (1:10 Receptor to compound) for 20 mins incubation on ice prior loading to NT.Plex nanoDSF Grade Capillaries (NanoTemper). Melting curves of tβ_1_AR were determined using Prometheus Melting Control v1.9 (NanoTemper) by measuring intrinsic protein fluorescence signal and its change during a temperature ramp from 20 to 95 °C at rate 2 °C/min. The melting temperature of receptor was measured in triplicates and an average melting temperature was obtained.

### Hydrogen deuterium exchange mass spectrometry (HDX-MS) for purified tβ_1_AR

The equilibration buffer (E) was composed of 20mM Tris-HCl, pH=8, 0.35 M NaCl, 3 mM imidazole, 0.05% DDM. The quench buffer (Q) was composed of 50 mM K_2_HPO_4_, 50 mM KH_2_PO_4_, 0.1% DDM, 100 mM TCEP. The labelling buffer (L) has the same composition as buffer E except H_2_O was substituted with D_2_O (99.8%). For the conditions of drug treatment, 300 μM of drug was preincubated with the protein samples prior deuterium labelling. Deuterium labelling was performed by diluting 5 μl of protein at concentration 16 μM in 95 μL of buffer L. The protein sample was incubated for various time points and then quenched with buffer Q at 1 °C and a pH of 2.3. Samples were immediately digested with a pepsin column conjugated with a HPLC system. For peptide analysis, HPLC run time was 11 min at flow rate of 40 μl/min under a gradient between buffer A (0.1% formic acid in H_2_O) and buffer B (acetonitrile with 0.1% formic acid). The columns used during the experiment was C18 trap (ACQUITY UPLC®BEH 1.7 mm, Waters), a C18 column (ACQUITY UPLC®BEH, 1,7 mm, 1.0 x 100 mm, Waters). The mass range for MS was m/z 100-2000 in positive ion mode on the Synapt G2-Si mass spectrometer with ESI source and ion mobility cell, coupled to ACQUITY UPLC with HDX Automation technology (Waters Corporation, Manchester, UK). The HDX analysis was performed at 4 time points (15 sec, 2, 30 and 120 min). Clean blank was injected between each analytical injections in order to remove carryover. The data for each time point were obtained in three replicates. The data were processed and analysed using MassLynx v4.1 (Waters), PLGS (ProteinLynx Global Server) used to analyse the MS data of unlabelled peptide sand generate peptide libraries for each target protein. DynamX 3.0 (Waters) used to analyse and quantify the deuteration for each peptide and Deuteros 2.0 used to sort out statistically significant differences in deuterium uptake for peptides in two different conditions. The HDX results for each of the ligand bound to tβ_1_AR were mapped onto the published structure (PDB 2YCW).

### Inductively coupled plasma mass-spectrometry (ICP-MS) analysis

tβ_1_AR, mini-G_s_ and their respective buffers were digested in digest on a hotplate using 0.3 molar HNO_3_. The samples were analysed for trace element concentrations using a PerkinElmer NexION 350D quadrupole inductively coupled plasma mass-spectrometer. Each element was calibrated from a series of calibration standards, which were robotically prepared by an Elemental Scientific prepFAST M5 autosampler. The stock standards were freshly prepared from a collection of synthetic ICP elemental standards (Merck Certipur- single element and custom blend) and were diluted into 2% v/v HNO_3_. The ICP-MS was setup to measure a selection of elements together in one single method using the PerkinElmer Syngistix ICP-MS software. This method also adopted the use of the instrument’s dynamic reaction/collision cell: a technology that is designed to suppress molecular interferences and improve detection and accuracy for many elements.

### cAMP accumulation assay

Chinese Hamster Ovary (CHO) from Merck (85051005) maintained in DMEM/F12 cell culture media supplemented with 10% FBS and 1% L-glutamine, were grown to 70-80% confluence before transfection of engineered tβ_1_AR using FuGENE® HD (Promega) according to manufacturer’s instructions. The next day, CHO cells transiently expressing engineered tβ_1_AR were prepared as a cell suspension in assay buffer (HBSS containing 5mM HEPES, pH7.4, further supplemented with 0.1% w/v BSA and 500 mM IBMX), before being incubated with a range of concentrations of β-adrenoceptor ligands noradrenaline, isoprenaline, carmoterol, dobutamine, salbutamol, cyanopindolol and carazolol, negative control (assay buffer) and positive control (10 mM isoprenaline, 3 mM forskolin) conditions for 1 h at room temperature. After 1 h, cAMP levels were measured using the HTRF cAMP G_s_ HiRange kit (CisBio) according to manufacturer’s instructions with FRET levels being detected on a PHERAstar plate reader (BMG) via BMG Reader Control software v5.7 and FRET ratio calculations being performed using the plate reader embedded BMG MARS v4.01 software. All other data analysis was carried out using GraphPad Prism 8 (GraphPad), including the conversion of FRET ratios to cAMP levels from a cAMP standard curve constructed in the same experiment.

### Molecular dynamics simulations

The coordinates of inactive and active states of tβ_1_AR were taken from PDB 4BVN and 6H7N respectively. The coordinate of the active tβ_1_AR in complex with mini-G_s_ was constructed by combining the active tβ_1_AR (PDB 4BVN) with the mini-G_s_ in the A_2A_R-mini-G_s_ complex (PDB 5G53) via aligning β_1_AR to A_2A_R. The missing ICL3 was stabilized by connecting the end residues D242 and S273. The protein structures were placed in a 10 × 10 nm^2^ membrane containing 80% POPC and 20% CHOL via CHARMM-GUI^37^, and then solvated with TIP4P waters with margins of 1.5 nm from the proteins. The systems were then neutralized by 150 mM NaCl and added 0.35 mM ZnCl_2_. Three replicas were constructed for each conformational state with different membrane configurations. The MD simulations were performed using GROMACS 2018 package^38^, using CHARMM 36 force field for proteins^39^ and lipids^40^. The LINCS^41^ method was used to restrain all bonds, allowing for a save integration of 2 fs. Lennard-Jones and Coulomb cut-off distances were set to 1.2 nm and the neighbour search cutoff was set to 1.2 nm with an update frequently of 10 fs. Particle mesh Ewald method was used to treat long range electrostatic interactions.

Starting configurations were subjected to steepest minimization to remove close contacts. The systems were then slowly heated to 303 K using an NVT ensemble with V-rescale thermostat. After that, a 10-ns equilibration was performed for each system using NPT ensemble in which the pressure was kept constant at 1 bar by semi-isotropic coupling to a Parrinello-Rahman barostat with τ_P_ = 5.0 ps and a compressibility of 4.6 x 10^-5^ bar whereas the temperature was maintained at 303 K by coupling (t_T_ = 0.5 ps) the protein membrane and solvent to a Nose-Hoover thermostat. Throughout the heating and equilibration process, a harmonic position restraint was added on protein back bone atoms and lipid headgroups. The production run used the same parameters as the equilibration step except for the positional restraints. 500 ns of simulation data was collected from each simulation replica.

The Zn^2+^ binding sites were calculated from the simulation data via PyLipID (github.com/wlsong/PyLipID). The binding sites were identified by community structures of the network, that is groups of nodes that are more densely connected internally than with the rest of the network. Zn^2+^ binding sites were calculated respectively from the inactive β_1_AR, active β_1_AR and the active β_1_AR in complex with mini-G_s_ simulations. The binding sites whose Zn^2+^ residence time showed prominent increase from the inactive simulations to the active or active complex with mini-G_s_ simulations.

To study the effect of Zn^2+^ on the association between β_1_AR and mini-G_s_, we calculated the potential of mean force (PMF) of mini-G_s_ dissociation from β_1_AR in the presence and absence of ZnCl_2_. The final system snapshot was taken from one replica of β_1_AR-mini-G_s_ simulations. For the calculation of PMF in the absence of ZnCl_2_, zincs and chlorides were taken out from the systems and then additional equilibration was performed to the systems. For generating configurations for umbrella samplings, Steered MD was carried out to pull mini-G_s_ away from β_1_AR along the z axis (perpendicular to the membrane plane). The distance between the centre of mass of β_1_AR and H5 motif of mini-G_s_ was monitored to ensure a pulling speed of 0.1nm/ns with a force constant of 1000 kJ/(mol nm^2^). The starting configurations of the umbrella sampling were extracted from SMD trajectories with spacing of 0.1 nm along the monitored distance. 35 windows were generated, and each collected 300 ns simulation data. The PMF was extracted from the umbrella sampling using the Weighted Histogram Analysis Method (WHAM) provided by the GROMACS *g_wham* tool. A Bayesian bootstrap was used to estimate the statistical error of the energy profile.

## Supplementary Material

Binding stoichiometry of the metal ligand in the intermediate and stable complex of tβ1AR-min

extent of inhibition for different Zn contact mutants

ICP MS

intensities of the metal ion bound species

plot of %

plot of normalised intensity for Mini-Gs coupling and adduct binding

Relative percentage of stoichiometric distribution of tβ1AR monomer and the E392A mutant

Relative percentage of stoichiometric distribution of tβ1AR monomer and tβ1AR-mini-Gs

text file 1

text file 2

Tm values for complexes formed in the presence of isoprenaline derivatives

Supplementary Material

## Data Availability

Data supporting the main conclusions of this study are in the Article, Supporting information and Source Data. Additional data that support the findings of this study are available as follows: native MS data and HDX-MS data have been deposited at Figshare with the following links: https://doi.org/10.6084/m9.figshare.19688640 and https://doi.org/10.6084/m9.figshare.19754662.v1
