# Supplementary material for: Mass spectrometry captures biased signaling and allosteric modulation of a G protein-coupled receptor
Source: Nat Chem. Author manuscript; Available in PMC 2022 Dec 20. (PMC9758051; doi:10.1038/s41557-022-01041-9)
Supplement: Supplementary Material [file EMS152486-supplement-Supplementary_Material.docx]

1. **Flat Files**

| Item | Present? | Filename | A brief, numerical description of file contents. |
| --- | --- | --- | --- |
| Supplementary Information | Yes | Supplementary Figures 1-10.pdf | Supplementary Figures 1-10 |
| Reporting Summary | Yes | New reporting summary pdf |  |
| Peer Review Information | No | *OFFICE USE ONLY* |  |

1. **Source Data**

| Parent Figure or Table | Filename | Data description |
| --- | --- | --- |
| Source Data Fig. 1 | Fig.1h.txt | Statistical source data |
| Source Data Fig. 3 | Fig.3c.txt Fig.3d.txt | Statistical source data |
| Source Data Fig. 4 | Fig.4d.txt | Statistical source data |
| Source Data Fig. 5 | Fig.5b_1.txt; Fig.5b_2.txt; Fig.5c.txt | Statistical source data |
| Source Data Extended Data Fig. 1 | Fig. S1a.txt | Statistical source data |
| Source Data Extended Data Fig. 4 | Fig.S4b.txt | Statistical source data |
| Source Data Extended Data Fig. 6 | Fig.S6.txt | Statistical source data |
| Source Data Extended Data Fig. 9 | Fig.S9d.txt | Statistical source data |
