# Supplementary material for: Mass spectrometry captures biased signalling and allosteric modulation of a G-protein-coupled receptor
Source: Nat Chem. 2022 Nov 10;14(12):1375–82. doi: 10.1038/s41557-022-01041-9 (PMC9758051; doi:10.1038/s41557-022-01041-9)
Supplement: Supplementary file 1 [file 41557_2022_1041_MOESM1_ESM.pdf]

nature portfolio

https://doi.org/10.1038/s41557-022-01041-9

Supplementary information

Mass spectrometry captures biased signalling and allosteric modulation of a G-protein-coupled receptor

In the format provided by the authors and unedited

Supplementary Information Materials for:

Mass spectrometry captures biased signaling and allosteric modulation of a G protein-coupled receptor

Hsin-Yung Yen[1,2]†*, Idlir Liko[1,2], Wanling Song[3]‡, Parth Kapoor[2], Fernando Almeida[2], Joanna Toporowska[4], Karolina Gherbi[2], Jonathan T. S. Hopper[2], Steven Charlton[2,5], Argyris Politis[4, ¥], Mark S. P. Sansom[3], Ali Jazayeri[2*,] Carol V. Robinson[1,6*]

Affiliations:
[1]Chemical Research Laboratory, University of Oxford; South Parks Road, OX1 3QY, U.K.
[2]OMass Therapeutics Ltd.; the Oxford Science Park, Heatley Rd, Oxford, OX4 4GE, U.K.
[3]Department of Biochemistry, University of Oxford; South Parks Road, OX1 3QU, U.K.
[4]Department of Chemistry, King's College London, 7 Trinity Street, London, SE1 1DB, UK
[5]School of Life Sciences, Queen's Medical Centre, University of Nottingham, NG7 2UH, UK
[6]Kavli Institute for Nanoscience Discovery, South Parks Road, Oxford, OX1 3QU, UK.

Present addresses:
†Institute of Biological Chemistry, Academia Sinica, 128, Academia Road Sec. 2, Nankang, Taipei 115, Taiwan
‡Rahko; Clifton House, 46 Clifton Terrace, Finsbury Park, London N4 3JP, UK
¥Faculty of Biology, Medicine and Health, School of Biological Sciences, The University of Manchester, Manchester, M13 9PT, UK.

This pdf includes:

Methods: pages 2 – 6

Supplementary Figures 1- 10: pages 7 - 16

Supplementary references: page 17

Methods:

Constructs and proteins. Expression plasmid for a thermostabilized variant of turkey (M. gallopavo) β_1AR was used in our experiments. The synthesized cDNA encoding tβ_1AR, flanked with N-terminal Flag tag and Strep tag, and C-terminal His tag, was cloned into pFastBac^{TM} 1 vector via NheI and NotI cloning sites. The protein sequence ranges from 44-364 with intracellular loop 3 (ICL3) truncation (244-272), and contains 7 thermostabilizing point mutations (R68S, C116L, E130W, D322K, F327A, F338M, C358A). Purified β_1AR, engineered Gα_s (mini-G_s) and Nanobody Nb6B9 were utilized for mass spectrometry analysis.

Expression and purification of mini-G_s and mini-$G_{i/s}$. The engineered minimal G protein, mini-G_s construct R414 and mini-$G_{i/s}$ construct R43 cloned into pET24a vector were expressed in *E. coli* BL21(DE3) strain and purified by Ni^{2+} affinity chromatography, followed by cleavage of the histidine tag using TEV protease. The cleaved tag, protease and undigested mini-G proteins were removed by reverse IMAC purification on Ni^{2+}-NTA. Proteins were concentrated to 2 mg/ml in 20 mM HEPES, pH 7.5, 100 mM NaCl, 10% v/v glycerol, 1 mM $MgCl_2$, and 10 mM GDP.

Expression and purification of tβ_1AR. The construct of tβ_1AR was over-expressed in insect cells using Bac-to-Bac® Baculovirus expression system (Thermo Fisher). The recombinant baculoviruses prepared using the expression vector pFastBac 1 (Thermo Fisher) were applied to infect Sf9 cells (Invitrogen, 11496015) with the Multiplicity of Infection (MOI) between 1-2.The cell membrane was enriched and solubilized in 20 mM Tris-HCl pH8, 350 mM NaCl, 3 mM imidazole, 1.5% (w/v) n-dodecyl-β-D-maltopyranoside (DDM, Anatrace) for 15 mins. The supernatant was isolated by ultra-centrifugation at 175,000 x g for 1 hr and applied to HiTrap TALON crude column (GE healthcare) for affinity enrichment. The column was washed by ten column volumes of 20 mM Tris–HCl pH 8, 350 mM NaCl, 3 mM imidazole and 0.05% DDM after loading the supernatant, and receptor was eluted by a gradient of 20 mM Tris–HCl pH 8, 350 mM NaCl, 250 mM imidazole and 0.05% DDM in three column volumes. The pH of all buffers were adjusted at room temperature. The fractions containing receptor were pulled and concentrated to the final concentration 2-3 mg/ml via Amicon® centrifugal filter of molecular weight cut-off 50 kDa for following applications. In order to mitigate the experimental variations which may cause the differential binding with endogenous zinc ions, we carefully controlled our experimental conditions during purification of the various tβ_1AR mutants. Specifically, the quantity of starting biomass (20 g), ratio between cell membranes and detergents (DDM: membrane proteins = 3:1 (w/w)), duration of detergent solubilization (15 mins) and FPLC conditions were strictly controlled.

Expression and purification of nanobody Nb6B9. The expression gene of Nb6B9 was cloned into the plasmid pET-26b(+)[34] which contains a N-terminal His-tag followed by a thrombin protease cleavage site. Protein was overexpressed in E. coli strain BL21(DE3) (Agilent Technologies) and purified from the periplasmic fraction was by Ni^{2+} affinity chromatography. The His-tag was removed with the use of a thrombin protease (Sigma) before concentration to 20 mg/ml.

Non-denatured mass spectrometry of tβ_1AR. Purified β_1AR was buffer exchanged into 200 mM ammonium acetate buffer pH 7.4 containing the mixed micelle preparation (DDM:

Foscholine16: CHS = 20: 2: 3 (w/w/w)) optimized for GPCR analysis as described previously[8] before MS analysis by a modified Q-Exactive mass spectrometer (Thermo)[35]. The capillary voltage (1.1 kV) was applied during nano-electrospray, and an optimized acceleration voltage (120 V) was then applied to the HCD cell to remove the detergent micelle from the protein ions, following a gentle voltage gradient (injection flatapole, inter-flatapole lens, bent flatapole, transfer multipole: 7.9, 6.94, 5.9, 4 V respectively). For analyzing receptor complex formation with mini-G_s, the optimized voltage was applied to the in-source fragmentation (100 V) and HCD cell (100 V) with the same voltage gradient for ion transmission. Spectra were acquired and averaged with a noise level parameter of 3. Backing pressure was maintained at ~0.9 x 10^{-9} mbar. Data was analyzed using Xcalibur 2.2 and the relative percentage of tβ_1AR in different binding stoichiometry was quantified by UniDec software[36]. The measurement error was derived from the deviation of peak centroids of different charge states corresponding to the same mass species.

Mini-G_α and Nb6B9 coupling to tβ_1AR. Effector coupling to tβ_1AR was analyzed by a modified Q-Exactive mass spectrometer after incubating purified tβ_1AR with mini-G_α/Nb6B9 at 1:1.2 molar ratio at 4 °C in the coupling buffer (10 mM HEPES, 10 mM Tris-HCl, pH7.4, 200 mM NaCl, 1mM $MgCl_2$, 5 mM GDP and 0.05% DDM) containing 25 μM agonists for at least 20 mins. To strip the exogenous metal ligand, both purified tβ_1AR and mini-G_s were pre-treated with 5mM EDTA for 5 mins at 4 °C and then buffer-exchanged into EDTA-free buffers for tβ_1AR (20 mM Tris–HCl pH 8, 350 mM NaCl and 0.05% DDM) and mini-G_s (20 mM HEPES, pH 7.5, 100 mM NaCl, 1 mM $MgCl_2$, and 10 mM GDP), respectively. The relative percentage of effector coupling was quantified by UniDec software and the degree of effector coupling was calculated by normalizing the relative percentage of complex to the sum of the percentage of receptor monomer and complex. To examine the inhibitory effect of antagonist, purified tβ_1AR was pre-incubated with carazolol at desired concentration for 10 mins at 4 °C, followed by the same procedure described above for mini-G_s coupling in the presence of isoprenaline. All data analysis was carried out using GraphPad Prism 7 (GraphPad).

To investigate the intermediate complex formation, purified tβ_1AR and mini-G_s were buffer-exchanged into 200 mM ammonium acetate buffer pH 7.4 containing the mixed micelle preparation and 5 mM GDP. tβ_1AR was pre-mixed with mini-G_α at 1:1 molar ratio at 4°C and the protein mixture was introduced into mass spectrometry immediately after adding isoprenaline to final concentration 25 mM. Spectra were acquired for 1 min and the relative percentage of tβ_1AR monomer, tβ_1AR-mini-G_s intermediate and stable complexes was quantified by UniDec software.

Nano differential scanning fluorimetry (nanoDSF) for stability measurement of purified tβ_1AR. tβ_1AR was diluted to 0.4 mg/ml in protein buffer (20 mM Tris–HCl pH 8, 0.35M NaCl, 3 mM imidazole and 0.05% DDM). β_1AR compounds such as agonists, agonist derivatives, antagonists and partial agonists were tested at concentrations 100 mM to measure their impact on the receptor stability. The DMSO concentration was maintained at 5% in the final reaction volume, and the control experiments including protein alone, protein in 5% DMSO and compounds alone were conducted as the appropriate references for measuring stabilization effect of compounds. Protein sample and compounds were mixed at a fixed molar ratio (1:10 Receptor to compound) for 20 mins incubation on ice prior loading to NT.Plex nanoDSF Grade Capillaries (NanoTemper). Melting curves of tβ_1AR were determined using Prometheus Melting Control

v1.9 (NanoTemper) by measuring intrinsic protein fluorescence signal and its change during a temperature ramp from 20 to 95 ^{o}C at rate 2 ^{o}C/min. The melting temperature of receptor was measured in triplicates and an average melting temperature was obtained.

Hydrogen deuterium exchange mass spectrometry (HDX-MS) for purified tβ_1AR. The equilibration buffer (E) was composed of 20mM Tris-HCl, pH=8, 0.35 M NaCl, 3 mM imidazole, 0.05% DDM. The quench buffer (Q) was composed of 50 mM K_2HPO_4, 50 mM KH_2PO_4, 0.1% DDM, 100 mM TCEP. The labelling buffer (L) has the same composition as buffer E except H_2O was substituted with D_2O (99.8%). For the conditions of drug treatment, 300 μM of drug was preincubated with the protein samples prior deuterium labelling. Deuterium labelling was performed by diluting 5 μl of protein at concentration 16 μM in 95 μL of buffer L. The protein sample was incubated for various time points and then quenched with buffer Q at 1 ^{o}C and a pH of 2.3. Samples were immediately digested with a pepsin column conjugated with a HPLC system. For peptide analysis, HPLC run time was 11 min at flow rate of 40 μl/min under a gradient between buffer A (0.1% formic acid in H_2O) and buffer B (acetonitrile with 0.1% formic acid). The columns used during the experiment was C18 trap (ACQUITY UPLC®BEH 1.7 mm, Waters), a C18 column (ACQUITY UPLC®BEH, 1,7 mm, 1.0 x 100 mm, Waters). The mass range for MS was m/z 100- 2000 in positive ion mode on the Synapt G2-Si mass spectrometer with ESI source and ion mobility cell, coupled to ACQUITY UPLC with HDX Automation technology (Waters Corporation, Manchester, UK). The HDX analysis was performed at 4 time points (15 sec, 2, 30 and 120 min). Clean blank was injected between each analytical injections in order to remove carryover. The data for each time point were obtained in three replicates. The data were processed and analysed using MassLynx v4.1 (Waters), PLGS (ProteinLynx Global Server) used to analyse the MS data of unlabelled peptide and generate peptide libraries for each target protein. DynamX 3.0 (Waters) used to analyse and quantify the deuteration for each peptide and Deuteros 2.0 used to sort out statistically significant differences in deuterium uptake for peptides in two different conditions. The HDX results for each of the ligand bound to tβ_1AR were mapped onto the published structure (PDB 2YCW).

Inductively coupled plasma mass-spectrometry (ICP-MS) analysis. tβ_1AR, mini-G_s and their respective buffers were digested in digest on a hotplate using 0.3 molar HNO_3. The samples were analysed for trace element concentrations using a PerkinElmer NexION 350D quadrupole inductively coupled plasma mass-spectrometer. Each element was calibrated from a series of calibration standards, which were robotically prepared by an Elemental Scientific prepFAST M5 autosampler. The stock standards were freshly prepared from a collection of synthetic ICP elemental standards (Merck Certipur- single element and custom blend) and were diluted into 2% v/v HNO_3. The ICP-MS was setup to measure a selection of elements together in one single method using the PerkinElmer Syngistix ICP-MS software. This method also adopted the use of the instrument's dynamic reaction/collision cell: a technology that is designed to suppress molecular interferences and improve detection and accuracy for many elements.

cAMP accumulation assay. Chinese Hamster Ovary (CHO) from Merck (85051005) maintained in DMEM/F12 cell culture media supplemented with 10% FBS and 1% L-glutamine, were grown to 70-80% confluence before transfection of engineered tβ_1AR using FuGENE® HD (Promega) according to manufacturer's instructions. The next day, CHO cells transiently expressing engineered tβ_1AR were prepared as a cell suspension in assay buffer (HBSS containing 5mM HEPES, pH7.4, further supplemented with 0.1% w/v BSA and 500 mM

IBMX), before being incubated with a range of concentrations of β-adrenoceptor ligands noradrenaline, isoprenaline, carmoterol, dobutamine, salbutamol, cyanopindolol and carazolol, negative control (assay buffer) and positive control (10 mM isoprenaline, 3 mM forskolin) conditions for 1 h at room temperature. After 1 h, cAMP levels were measured using the HTRF cAMP Gs HiRange kit (CisBio) according to manufacturer's instructions with FRET levels being detected on a PHERAstar plate reader (BMG) via BMG Reader Control software v5.7 and FRET ratio calculations being performed using the plate reader embedded BMG MARS v4.01 software. All other data analysis was carried out using GraphPad Prism 8 (GraphPad), including the conversion of FRET ratios to cAMP levels from a cAMP standard curve constructed in the same experiment.

Molecular dynamics simulations. The coordinates of inactive and active states of tβ_1AR were taken from PDB 4BVN and 6H7N respectively. The coordinate of the active tβ_1AR in complex with mini-G_s was constructed by combining the active tβ_1AR (PDB 4BVN) with the mini-G_s in the A_{2A}R-mini-G_s complex (PDB 5G53) via aligning β_1AR to A_{2A}R. The missing ICL3 was stabilized by connecting the end residues D242 and S273. The protein structures were placed in a 10 x 10 nm^2 membrane containing 80% POPC and 20% CHOL via CHARMM-GUI[37], and then solvated with TIP4P waters with margins of 1.5 nm from the proteins. The systems were then neutralized by 150 mM NaCl and added 0.35 mM $ZnCl_2$. Three replicas were constructed for each conformational state with different membrane configurations. The MD simulations were performed using GROMACS 2018 package[38], using CHARMM 36 force field for proteins[39] and lipids[40]. The LINCS[41] method was used to restrain all bonds, allowing for a save integration of 2 fs. Lennard-Jones and Coulomb cut-off distances were set to 1.2 nm and the neighbour search cutoff was set to 1.2 nm with an update frequently of 10 fs. Particle mesh Ewald method was used to treat long range electrostatic interactions.

Starting configurations were subjected to steepest minimization to remove close contacts. The systems were then slowly heated to 303 K using an NVT ensemble with V-rescale thermostat. After that, a 10-ns equilibration was performed for each system using NPT ensemble in which the pressure was kept constant at 1 bar by semi-isotropic coupling to a Parrinello-Rahman barostat with τ_P = 5.0 ps and a compressibility of 4.6 x 10^{-5} bar whereas the temperature was maintained at 303 K by coupling (t_T = 0.5 ps) the protein membrane and solvent to a Nose-Hoover thermostat. Throughout the heating and equilibration process, a harmonic position restraint was added on protein back bone atoms and lipid headgroups. The production run used the same parameters as the equilibration step except for the positional restraints. 500 ns of simulation data was collected from each simulation replica.

The Zn^{2+} binding sites were calculated from the simulation data via PyLipID (github.com/wlsong/PyLipID). The binding sites were identified by community structures of the network, that is groups of nodes that are more densely connected internally than with the rest of the network. Zn^{2+} binding sites were calculated respectively from the inactive β_1AR, active β_1AR and the active β_1AR in complex with mini-G_s simulations. The binding sites whose Zn^{2+} residence time showed prominent increase from the inactive simulations to the active or active complex with mini-G_s simulations.

To study the effect of Zn^{2+} on the association between β_1AR and mini-G_s, we calculated the potential of mean force (PMF) of mini-G_s dissociation from β_1AR in the presence and absence of $ZnCl_2$. The final system snapshot was taken from one replica of β_1AR-mini-G_s simulations. For the calculation of PMF in the absence of $ZnCl_2$, zincs and chlorides were taken out from the systems and then additional equilibration was performed to the systems. For generating configurations for umbrella samplings, Steered MD was carried out to pull mini-G_s away from β_1AR along the z axis (perpendicular to the membrane plane). The distance between the centre of mass of β_1AR and H5 motif of mini-G_s was monitored to ensure a pulling speed of 0.1nm/ns with a force constant of 1000 kJ/(mol nm^2). The starting configurations of the umbrella sampling were extracted from SMD trajectories with spacing of 0.1 nm along the monitored distance. 35 windows were generated, and each collected 300 ns simulation data. The PMF was extracted from the umbrella sampling using the Weighted Histogram Analysis Method (WHAM) provided by the GROMACS *g_wham* tool. A Bayesian bootstrap was used to estimate the statistical error of the energy profile.

Supplementary figures and legends:

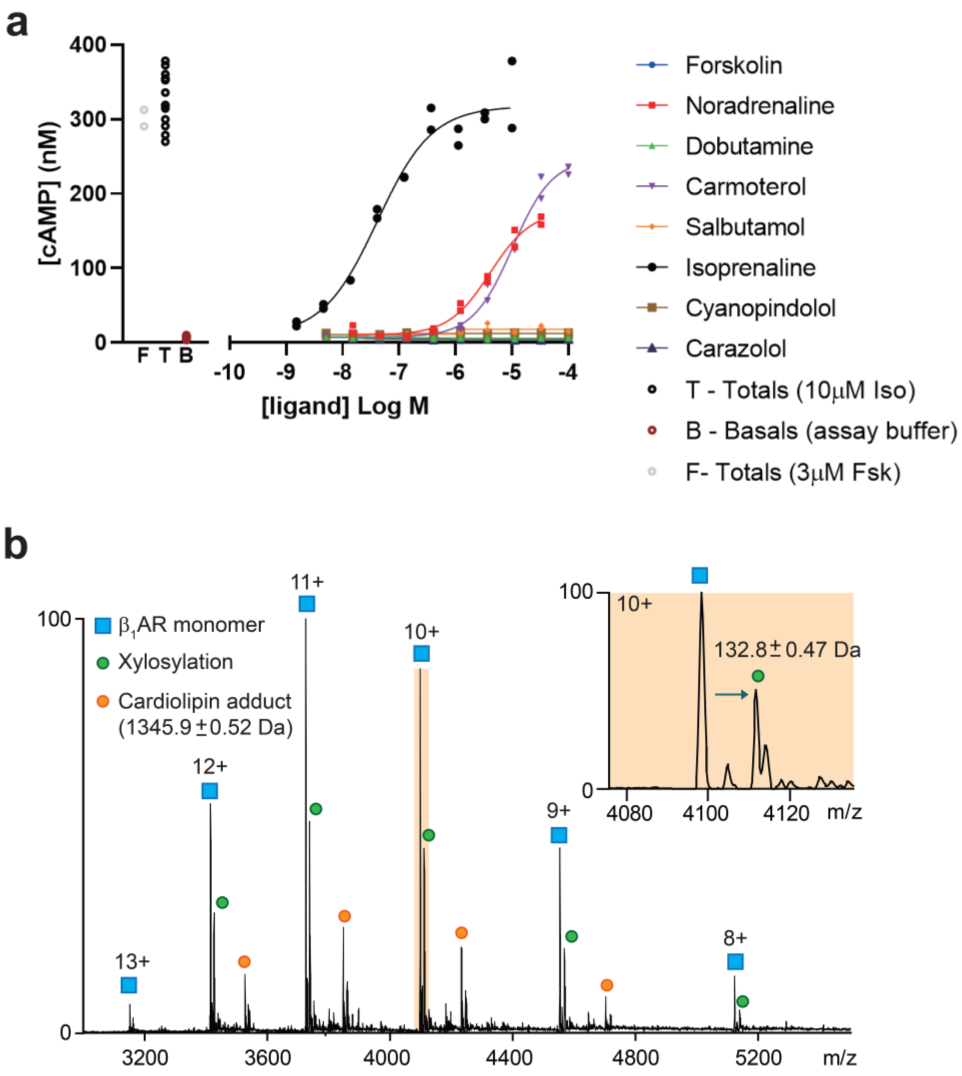

Fig. S1. Pharmacological and MS analysis of engineered β1AR. a, Ligand-induced cAMP production was measured in a cell line expressing engineered β1AR. Chinese Hamster Ovary (CHO) cells transiently expressing engineered β1AR were treated with increasing concentrations of noradrenaline, isoprenaline, carmoterol, dobutamine, salbutamol**,** cyanopindolol and carazolol. The intracellular cAMP levels in response to negative control (assay buffer) and positive controls (10 mM isoprenaline, 3 mM forskolin) were also determined. The curves are plotted as mean ± s.d. from three independent experiments. **b,** A representative MS spectrum of purified β1AR-E130W. In additional to receptor in apo state (blue square), a modification of 132.8 ± 0.47 Da was observed (green circle), suggesting O-xylosylation of receptor[42]. A cardiolipin adduct was detected with mass 1345.9 ± 0.52 Da (orange circle).

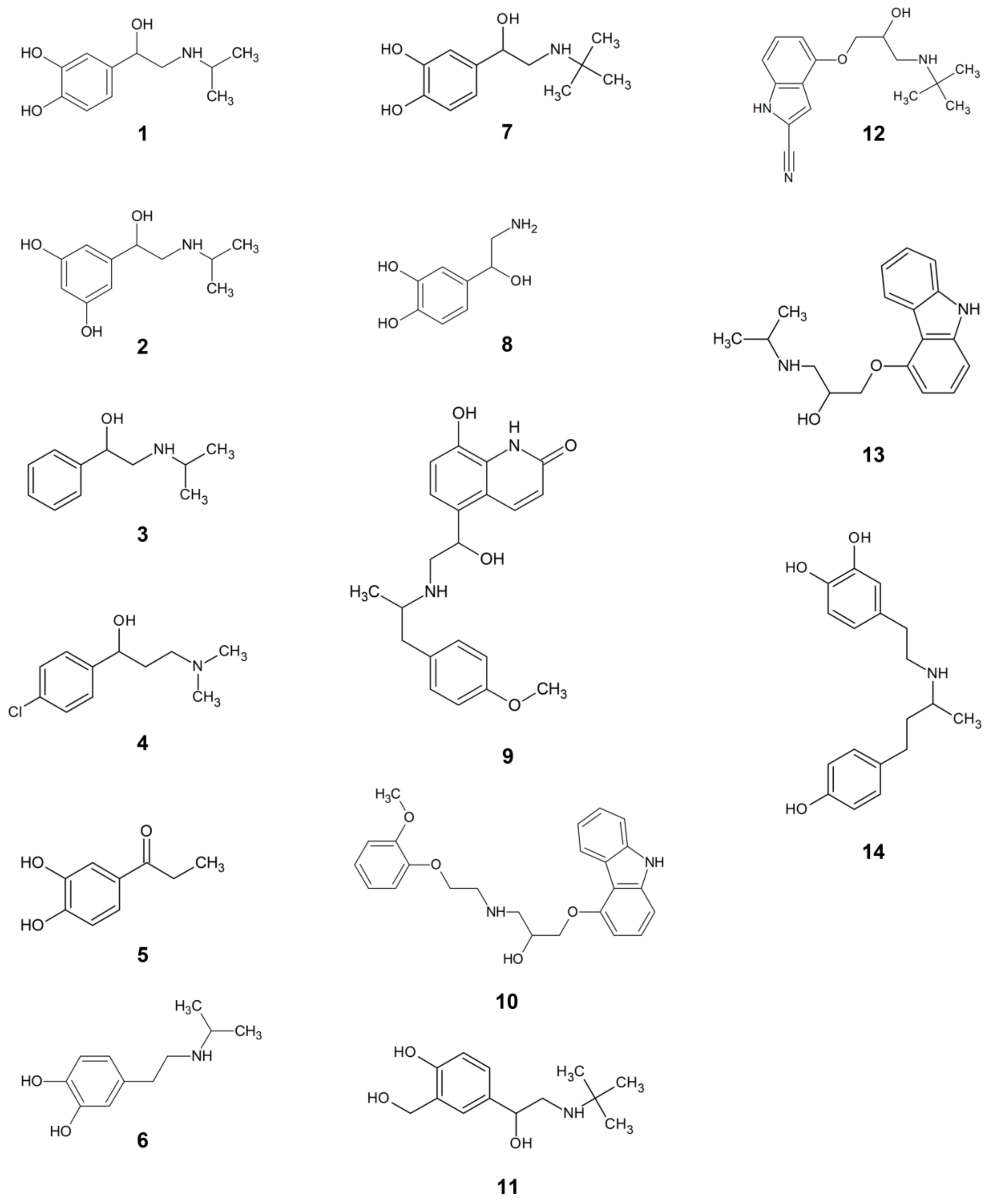

Fig. S2. Chemical structures of β1AR ligands. Isoprenaline derivatives: Isoprenaline **1**; Orciprenaline **2**; 1-phenyl-2[(propan-2-yl)amino]ethan-1-ol **3**; 1-(4-chlorophenyl)-3-(dimethylamino)propan-1-one hydrochloride **4**; 3,4 dihydroxypropiophenone **5**; Isopropyldopamin **6**; Colterol **7**. Full agonists: Norepinephrine **8**; Carmoterol **9**. Partial agonists: Dobutamine **10**; Salbutamol **11**. Antagonists: Cynopindolol **12**; Carazolol **13**; Carvedilol **14**.

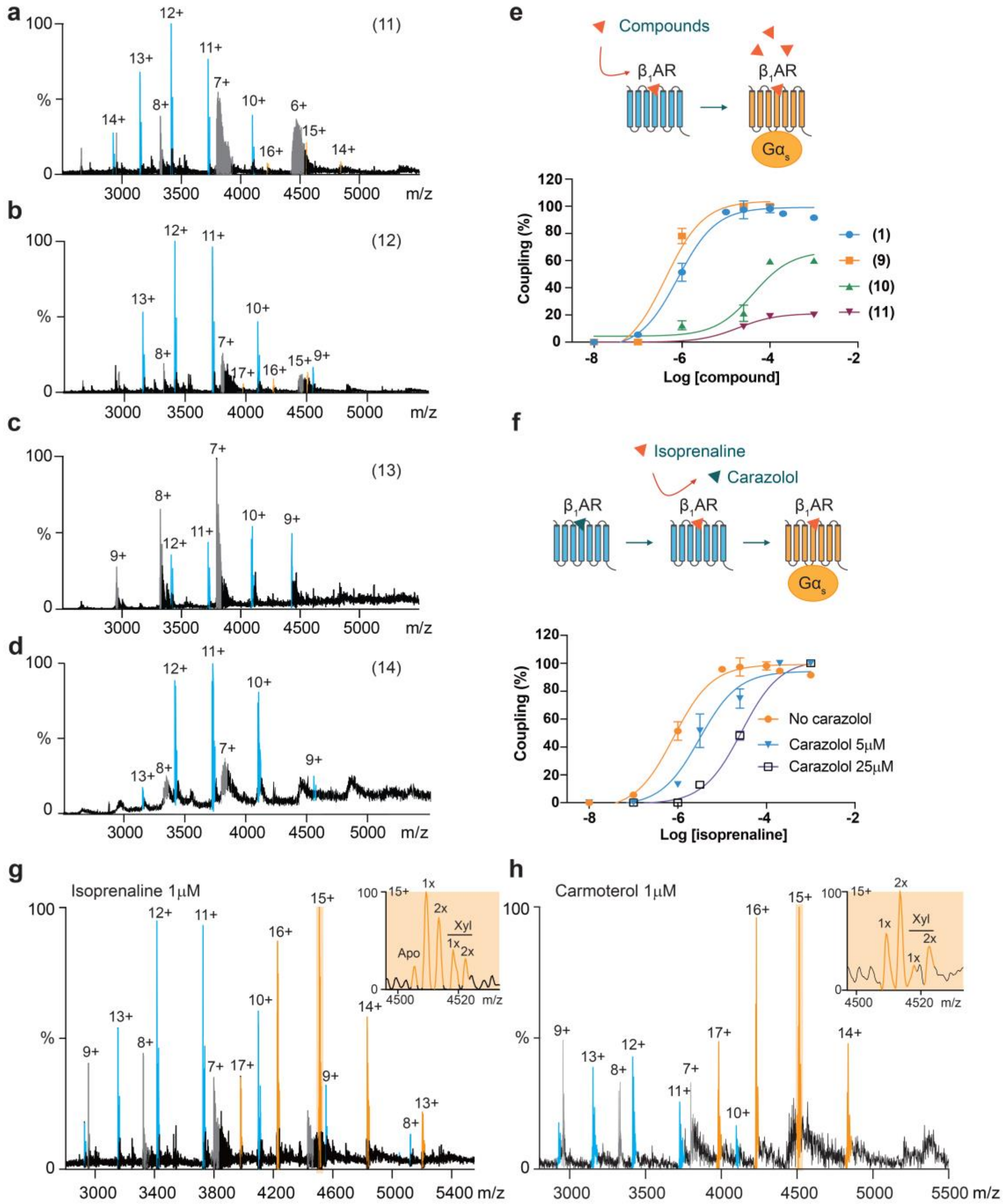

Fig. S3. Examination of mini-G_s-coupling of β_1AR with different ligands. The complex formation between β_1AR and mini-G_s was examined by native-MS under the stimulation of various compounds (Salbutamol **11**; Carazolol **13**; Carvedilol **14** at concentration 25 μM and Cyanopindolol **12** at concentration 100 μM). The peaks assigned to receptor-mini-G_s complex are highlighted in orange whereas receptor monomer and mini-G_s monomer is denoted in blue and grey, respectively. Representative spectrum from three independent experiments is shown. **e,** Dose-response curves of mini-G_s-coupling to the tβ_1AR agonists (Isoprenaline **1**; Carmoterol **9**; Dobutamine **10**; Salbutamol **11**). **f,** Response curves of mini-G_s-coupling to isoprenaline in the presence of carazolol at different concentrations. The curves in **e** and **f** are plotted as mean ± s.d. from three independent experiments.

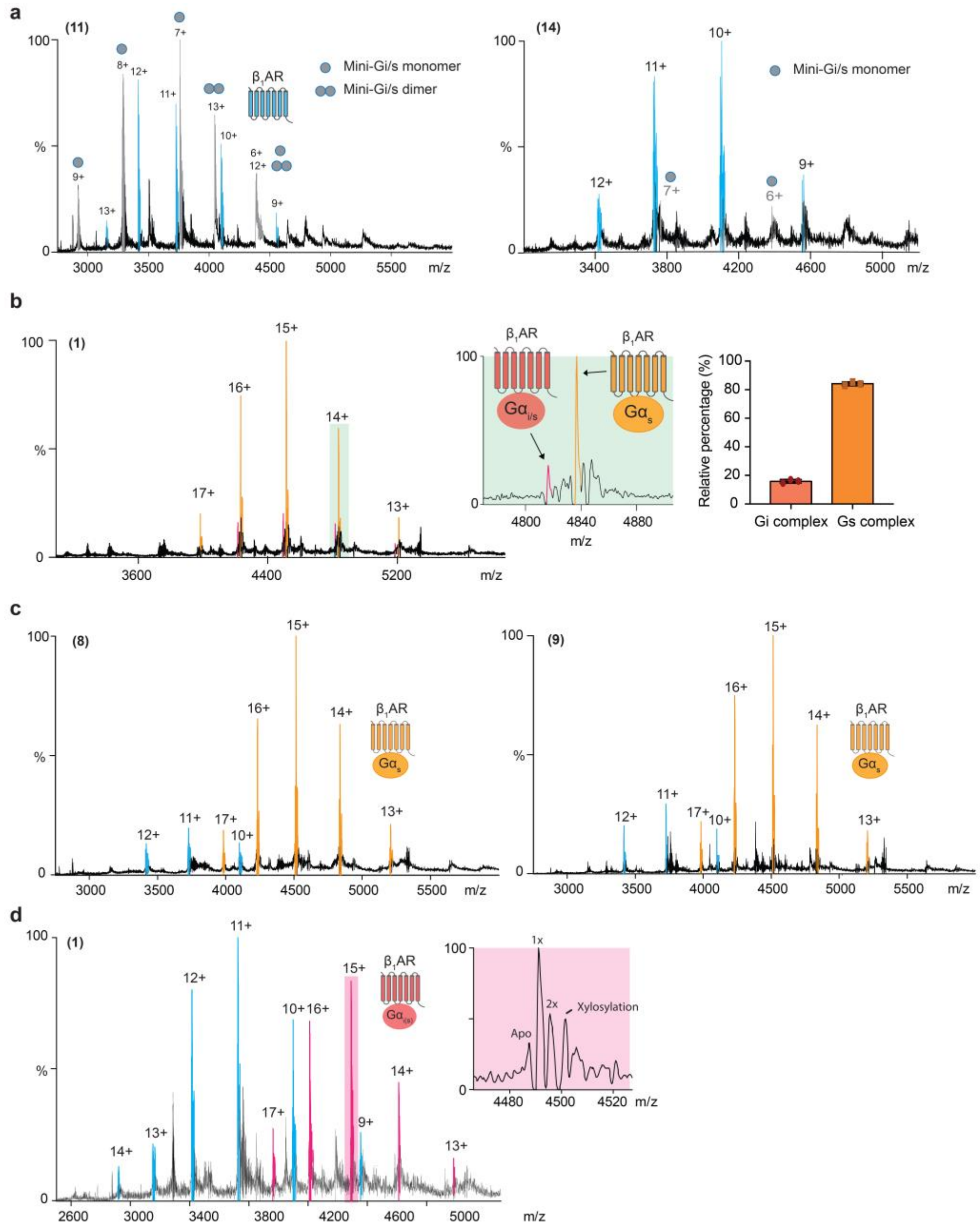

Fig. S4. Investigation of β_1AR-mini-G$_{i/s}$ complex formation in response to different agonists and metal-binding of the complex. a, No complex formation of β_1AR with mini-G$_{i/s}$ was detected with partial agonists salbutamol **11** and antagonist carvedilol **14**. **b,** Competition experiment for mini-G$_s$ and mini-G$_{i/s}$ binding to β_1AR (all at equimolar ratios). β_1AR-mini-G$_s$ and β_1AR-mini-G$_{i/s}$ complexes were both detected in the presence of isoprenaline **1** (green box). Bar chart normalised intensities of the individual to the total complexes (%) as a mean ± s.d. from three independent experiments. Full agonists norepinephrine **8** and carmoterol **9** are shown in **c** and **d,** β_1AR-mini-G$_{i/s}$ complex formation following stimulation with isoprenaline **1**. The binding stoichiometry of metal ions (15^+) highlighted (magenta box). Spectra from **a-d** are representative from three independent experiments. β_1AR-mini-G$_s$ and β_1AR-mini-G$_{i/s}$ complexes are highlighted (orange and magenta peaks respectively) receptor monomer (blue). Error bars are mean ± s.d. from three independent experiments (same batch of purified proteins).

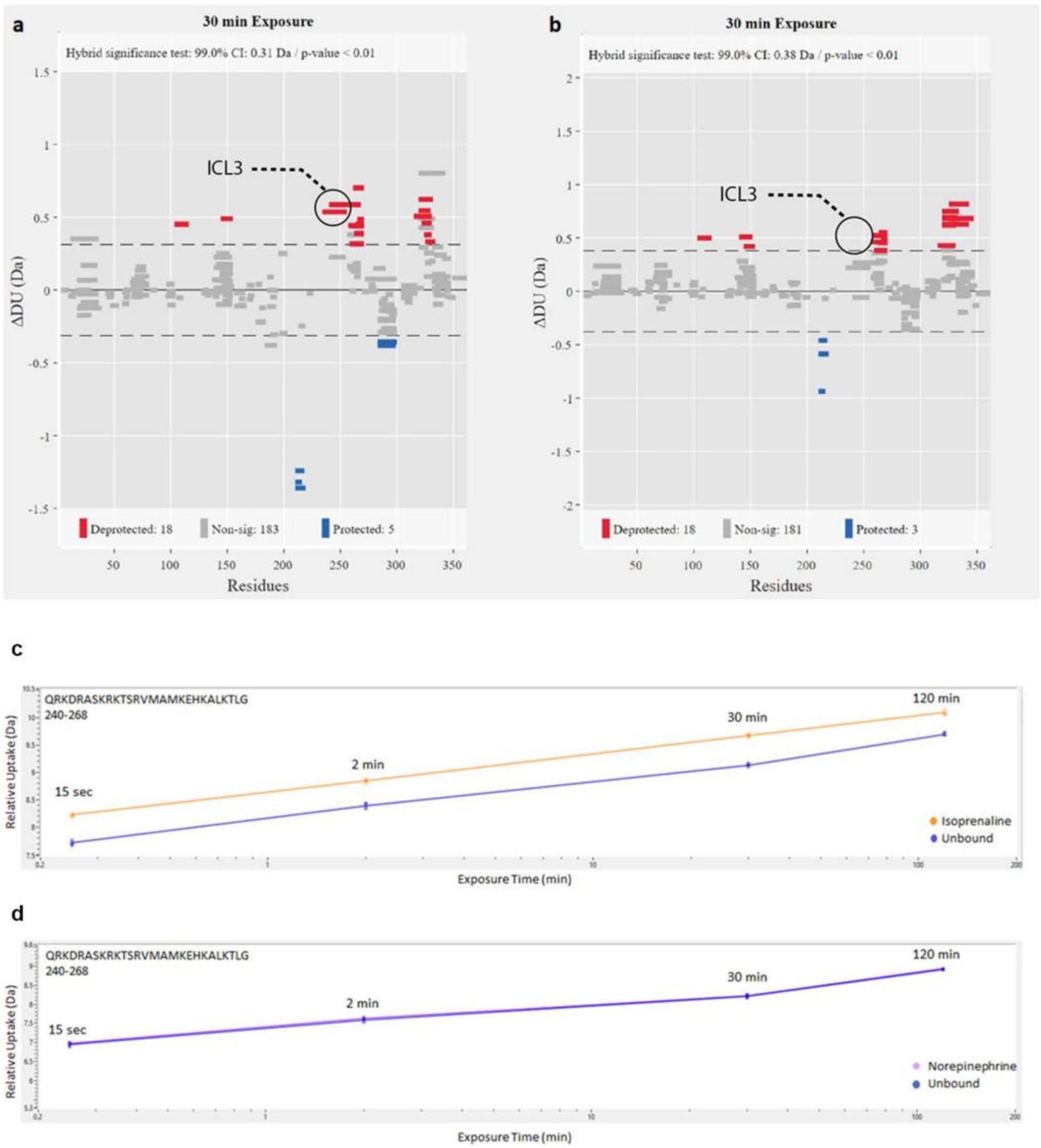

Fig. S5. Hydrogen deuterium exchange mass spectrometry (HDX-MS) analysis of β_1AR treated with isoprenaline or norepinephrine. Deuterium uptake differences between agonist-treated receptor and apo receptor are presented as Woods plot[43,44], illustrating the peptide ensemble of β_1AR as a function of global peptide coverage, peptide length and deuterium uptake difference ($DDu=Du^{drug}-Du^{apo}$). The deuterium uptake at a representative time point (30 min) was introduced, and a statistical analysis (99% confidence limit) was applied to identify peptides with significant difference in deuterium uptake for β_1AR bound to isoprenaline, **a**, and norepinephrine, **b,** in comparison to the apo state. Deprotected and protected peptides are coloured in red and blue, respectively. The ICL3 regions are highlighted (circled). The deuterium uptake plots of a peptide corresponding to β_1AR ICL3 (240-268) are shown in **c** and **d** for isoprenaline and norepinephrine, respectively, to illustrate the deuterium uptake of this peptide across four different labelling time points (15 sec, 2 min, 30 min and 120 min). Three independent experiments were performed and the bars shown in **c** and **d** represent mean ± s.d. of the triplicate from a representative experiment.

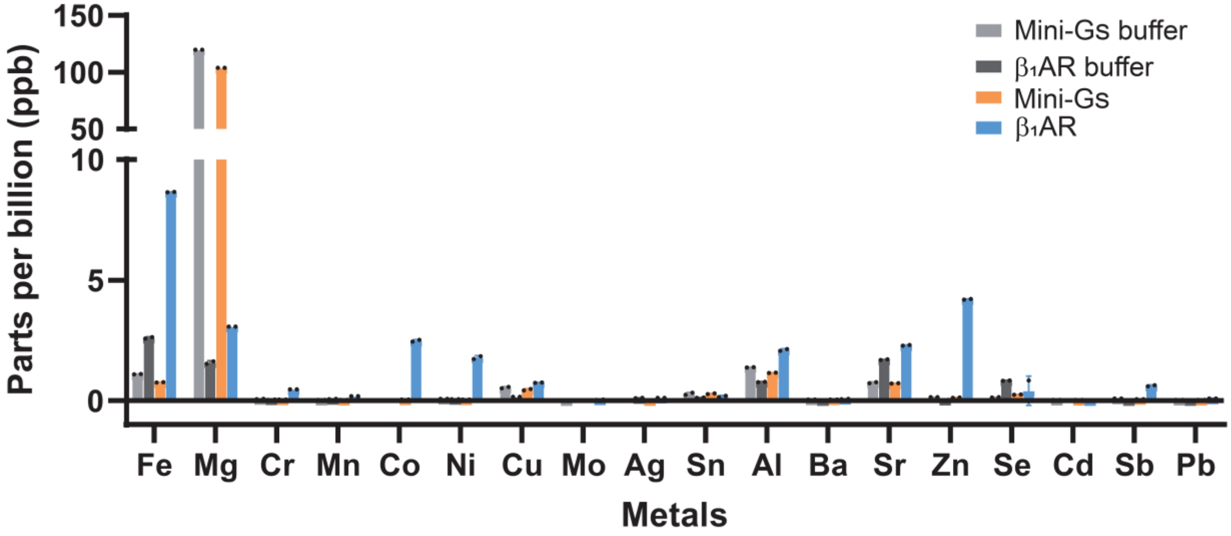

Fig. S6. Examination of trace elements by Inductively Coupled Plasma Mass Spectrometry (ICP-MS). The purified tβ_1AR and mini-G_s proteins were subjected to ICP-MS analysis, along with the buffers used for protein purification. Eighteen elements were examined and quantified through individual standards. The results revealed the presence of low amount of zinc only in solutions of tβ_1AR and no appreciable levels of copper. The results were determined from replicate experiments and error bars represent mean ± s.d.

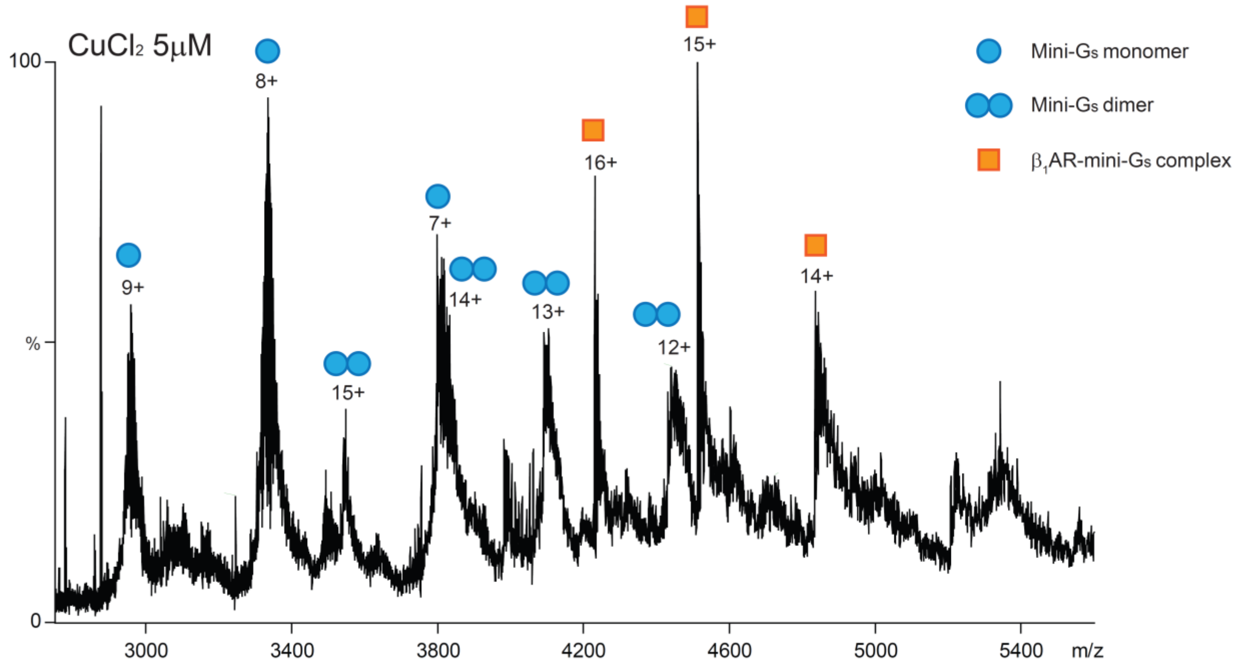

Fig. S7. Complex formation of β_1AR and mini-G$_s$ in the presence of copper. Peaks in the mass spectrum assigned to β_1AR-mini-G$_s$ complex are highlighted in orange, and mini-G$_s$ protein is denoted as blue circles. The observation of highly populated mini-G$_s$ in the uncomplexed state implies that copper does not significantly improve the coupling activity of the receptor. Three replicate experiments were performed.

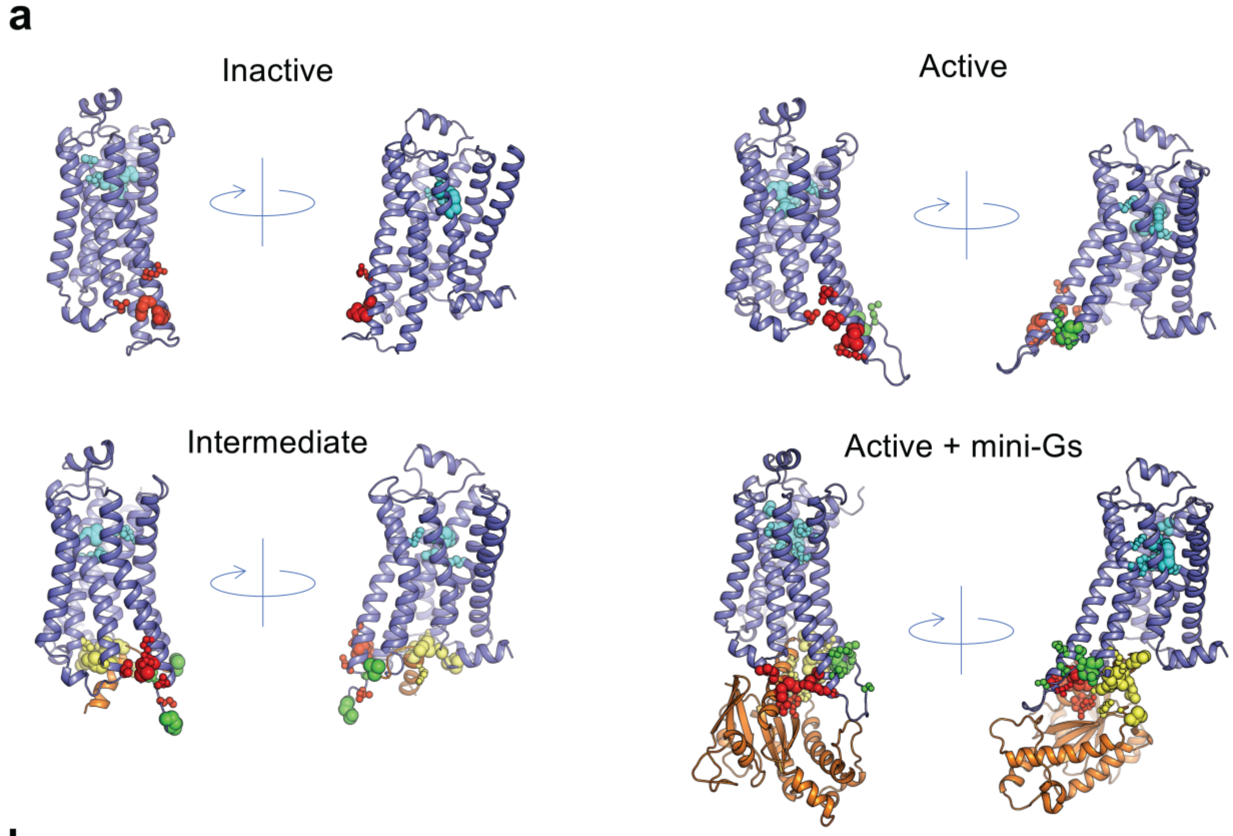

b

Zn^{2+} binding sites and Zn^{2+} residence times

Conf. State \ Binding Site	Orthosteric pocket	TM5-H5^{Gs}	TM6	H5^{Gs}
Inactive	500 ns	263 ns	None	None
Active	432 ns	303 ns	303 ns	None
Intermediate	500 ns	500 ns	500 ns	500 ns
Act + mini-Gs	465 ns	500 ns	356 ns	500 ns

Fig. S8. Zn^{2+} binding sites and Zn^{2+} residence times from molecular dynamics simulations. **a,** The Zn^{2+} binding sites on β1AR in the inactive (PDB 4BVN), active (PDB 6H7N), intermediate (H5 ^{Gs} bound) and mini-Gs coupled states were calculated from atomistic MD simulations. Contact residues for Zn^{2+} interactions at the orthostatic ligand binding site of the receptor are shown in cyan, at the intracellular interface of TM5 ^{β1AR} and H5^{Gs} are shown in red, at the intracellular end of TM6 ^{β1AR} are shown in green, and at the C terminal of H5^{Gs} are shown in yellow. All the binding site residues are shown in spheres with sphere scales corresponding to their Zn^{2+} residence times. **b,** Zn^{2+} residence times of the calculated binding sites on the four receptor conformations.

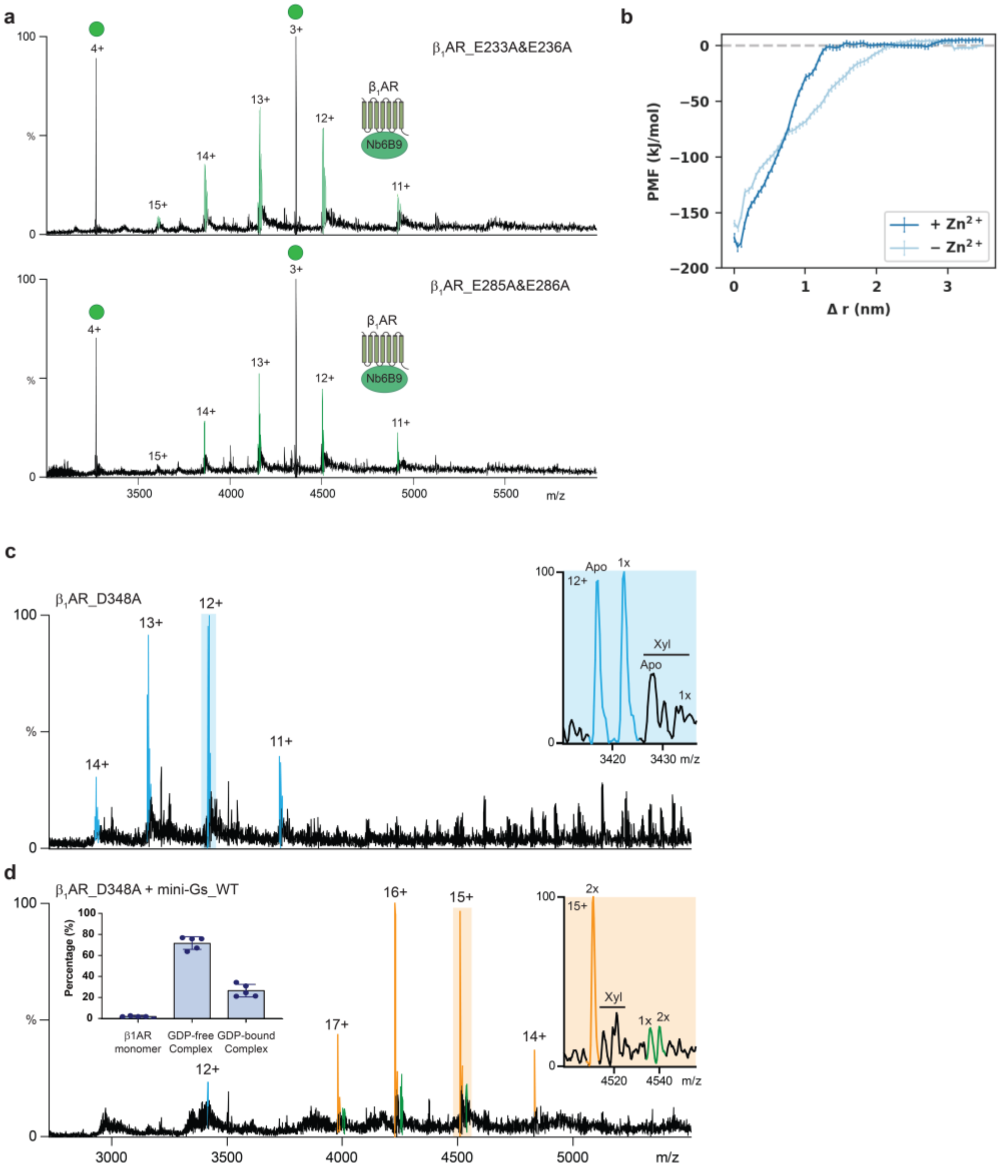

Fig. S9. Investigation of complex formation for zinc contact mutants of β_1AR and the free energy state of β_1AR-G$_s$ complex in the presence of zinc. a, MS of β_1AR variants with mutations on TM5 (E233A&E236A) and TM6 (E285A&E286A) with Nb6B9 complex (green) and free Nb6B9 (green circles). N = 3 independent experiments. **b,** Plot of potential mean force (PMF) for the interaction of mini-G$_s$ with β_1AR in the presence of zinc (dark blue) or in apo state (light blue). The PMF is calculated along a reaction coordinate (Δz) corresponding to the centre–centre separation of mini-G$_s$-β_1AR z axis (normal to the bilayer plane). Mini-G$_s$ -β_1AR is stabilized by ~15 kJ mol^{-1} in the presence of zinc. Error bars are from bootstrap sampling of the PMFs and represent statistical errors (mean ± s.d.) (n = 3 independent experiments). **c,** MS of β_1AR D348A shows high metal-binding to the receptor. **d,** MS of β_1AR D348A coupling to mini-G$_s$ in response to isoprenaline. The GDP-bound and GDP-free complex are highlighted (green and orange respectively) receptor monomer (blue). Relative quantification different stoichiometric states reveals a low percentage of receptor monomer compared with unmutated receptor (Fig. 4b, main text) (mean ± s.d. from five independent experiments from the same batch of purified receptor). Binding stoichiometry of metal ions is shown (orange box).

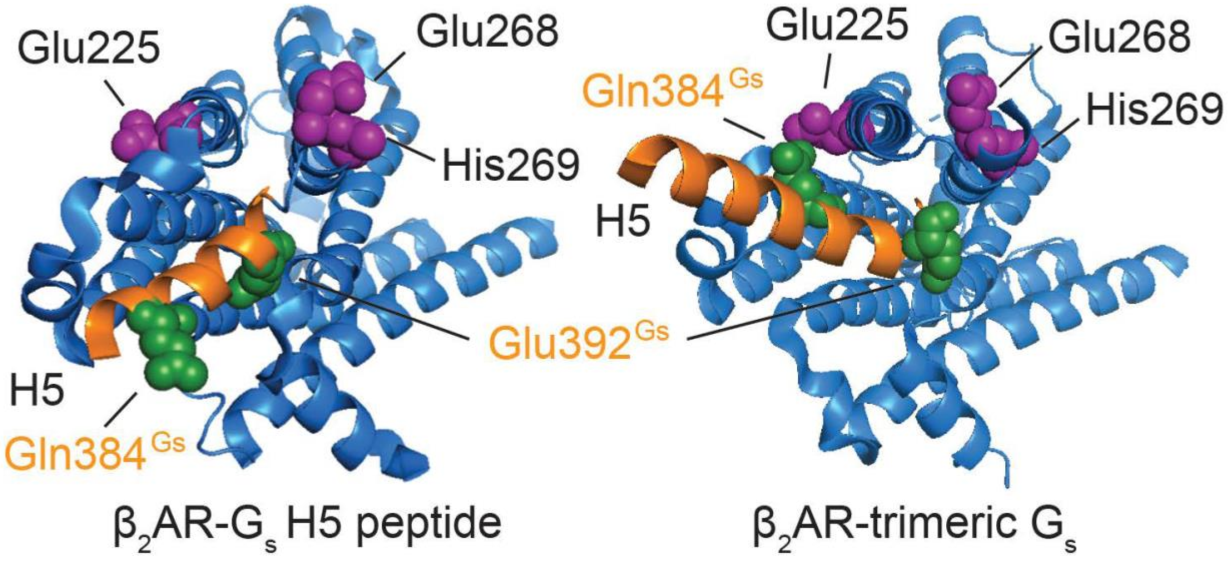

Fig. S10. Structure comparison of β$_2$AR-G$_s$ peptide and β$_2$AR-G$_s$ protein. Spatial orientations of G$_s$ H5 motif were highlighted in orange in the structures of β$_2$AR-G$_s$ H5 (PDB 6E67) peptide and β$_2$AR-trimeric G$_s$ protein (PDB 3SN6). The zinc contacting residues observed in this study are denoted as spheres on receptor (purple) and G$_s$ protein (green).

References for supplementary information:

42. Spencer, D. et al. O-xylosylation in a recombinant protein is directed at a common motif on glycine-serine linkers. J Pharm Sci 102, 3920-3924, doi:10.1002/jps.23733 (2013).

43. Woods, V. L., Jr. & Hamuro, Y. High resolution, high-throughput amide deuterium exchange-mass spectrometry (DXMS) determination of protein binding site structure and dynamics: utility in pharmaceutical design. J Cell Biochem Suppl Suppl 37, 89-98, doi:10.1002/jcb.10069 (2001).

44. Lau, A. M. C., Ahdash, Z., Martens, C. & Politis, A. Deuteros: software for rapid analysis and visualization of data from differential hydrogen deuterium exchange-mass spectrometry. Bioinformatics 35, 3171-3173, doi:10.1093/bioinformatics/btz022 (2019).
