# Supplementary material for: Mass spectrometry captures biased signalling and allosteric modulation of a G-protein-coupled receptor
Source: Nat Chem. 2022 Nov 10;14(12):1375–82. doi: 10.1038/s41557-022-01041-9 (PMC9758051; doi:10.1038/s41557-022-01041-9)
Supplement: Supplementary file 2 [file 41557_2022_1041_MOESM2_ESM.pdf]

nature research

Corresponding author(s): Hsin-Yung Yen, Ali Jazayeri, Carol V. Robinson

Last updated by author(s): Dec 10, 2021

Reporting Summary

Nature Research wishes to improve the reproducibility of the work that we publish. This form provides structure for consistency and transparency in reporting. For further information on Nature Research policies, see our Editorial Policies and the Editorial Policy Checklist.

Statistics

For all statistical analyses, confirm that the following items are present in the figure legend, table legend, main text, or Methods section.

n/a	Confirmed	
☐	☒	The exact sample size (n) for each experimental group/condition, given as a discrete number and unit of measurement
☐	☒	A statement on whether measurements were taken from distinct samples or whether the same sample was measured repeatedly
☒	☐	The statistical test(s) used AND whether they are one- or two-sided<br>*Only common tests should be described solely by name; describe more complex techniques in the Methods section.*
☒	☐	A description of all covariates tested
☒	☐	A description of any assumptions or corrections, such as tests of normality and adjustment for multiple comparisons
☐	☒	A full description of the statistical parameters including central tendency (e.g. means) or other basic estimates (e.g. regression coefficient) AND variation (e.g. standard deviation) or associated estimates of uncertainty (e.g. confidence intervals)
☒	☐	For null hypothesis testing, the test statistic (e.g. F, t, r) with confidence intervals, effect sizes, degrees of freedom and P value noted<br>*Give P values as exact values whenever suitable.*
☒	☐	For Bayesian analysis, information on the choice of priors and Markov chain Monte Carlo settings
☒	☐	For hierarchical and complex designs, identification of the appropriate level for tests and full reporting of outcomes
☒	☐	Estimates of effect sizes (e.g. Cohen's d, Pearson's r), indicating how they were calculated

Our web collection on statistics for biologists contains articles on many of the points above.

Software and code

Policy information about availability of computer code

Data collection	Xcalibur 2.2, MassLynx v4.1, Prometheus Melting Control v1.9, BMG Reader Control v5.7
Data analysis	UniDec, ProteinLynx Global Server, DynamX 3.0, Deuteros 2.0, Prometheus Melting Control v1.9, Prism 7, BMG MARS v4.01

For manuscripts utilizing custom algorithms or software that are central to the research but not yet described in published literature, software must be made available to editors and reviewers. We strongly encourage code deposition in a community repository (e.g. GitHub). See the Nature Research guidelines for submitting code & software for further information.

Data

Policy information about availability of data

All manuscripts must include a data availability statement. This statement should provide the following information, where applicable:

- Accession codes, unique identifiers, or web links for publicly available datasets
- A list of figures that have associated raw data
- A description of any restrictions on data availability

The raw HDX-MS data including all the deuterium uptake plots of the experiments presented for β1-AR are available on figshare data repository using the following link:https://doi.org/10.6084/m9.figshare.19754662.v1. Native MS data for all the results presented have been deposited to Figshare at https://doi.org/10.6084/m9.figshare.19688640.

Field-specific reporting

Please select the one below that is the best fit for your research. If you are not sure, read the appropriate sections before making your selection.

☒ Life sciences ☐ Behavioural & social sciences ☐ Ecological, evolutionary & environmental sciences

For a reference copy of the document with all sections, see nature.com/documents/nr-reporting-summary-flat.pdf

Life sciences study design

All studies must disclose on these points even when the disclosure is negative.

Sample size	No sample size calculations were performed. All proteins were selected based on underlining biology and availability.
Data exclusions	No data were excluded form the analysis.
Replication	All measurements were done at least in triplicate and all attempts at replication were successful and presented.
Randomization	There was no allocation in experimental groups involved.
Blinding	Blinding experiments were performed while examining the activity of isoprenaline derivatives and their stabilization effect to avoid the biased interpretations.

Reporting for specific materials, systems and methods

We require information from authors about some types of materials, experimental systems and methods used in many studies. Here, indicate whether each material, system or method listed is relevant to your study. If you are not sure if a list item applies to your research, read the appropriate section before selecting a response.

Materials & experimental systems

n/a	Involved in the study	
☐	☒	Antibodies
☐	☒	Eukaryotic cell lines
☒	☐	Palaeontology and archaeology
☒	☐	Animals and other organisms
☒	☐	Human research participants
☒	☐	Clinical data
☒	☐	Dual use research of concern

Methods

n/a	Involved in the study	
☒	☐	ChIP-seq
☒	☐	Flow cytometry
☒	☐	MRI-based neuroimaging

Antibodies

Antibodies used	Nanobody Nb6B9 were expressed and purified in E.coli strain BL21(DE3)RIL (Agilent Technologies).
Validation	Synthetic genes utilized in this study were validated previously as shown elsewhere Bang 2015 and Rasmussen 2011.

Eukaryotic cell lines

Policy information about cell lines

Cell line source(s)	Sf9 cells were obtained from Invitrogen; Chinese Hamster Ovary (CHO cells) were obtained from Merck.
Authentication	The Sf9 cell line was authenticated by the supplier by morphology, whereas CHO cells were .authenticated by STR profiling. None of the cell line used have been authenticated by the authors.
Mycoplasma contamination	Cell lines were tested negative for mycoplasma contamination.
Commonly misidentified lines (See ICLAC register)	No commonly misidentified cell lines were used.
